# Microarray profiling predicts early neurological and immune phenotypic traits in advance of CNS disease during disease progression in *Trypanosoma*. *b*. *brucei* infected CD1 mouse brains

**DOI:** 10.1371/journal.pntd.0009892

**Published:** 2021-11-11

**Authors:** Paul Montague, Barbara Bradley, Jean Rodgers, Peter G. E. Kennedy

**Affiliations:** 1 College of Medical, Veterinary and Life Sciences, Institute of Infection, Immunity and Inflammation, Glasgow, United Kingdom; 2 Institute of Biodiversity, Animal Health and Comparative Medicine, Glasgow, United Kingdom; University of Texas Southwestern Medical School, UNITED STATES

## Abstract

Human African trypanosomiasis (HAT), also known as sleeping sickness, is a major cause of mortality and morbidity in sub-Saharan Africa. We hypothesised that recent findings of neurological features and parasite brain infiltration occurring at much earlier stages in HAT than previously thought could be explained by early activation of host genetic programmes controlling CNS disease. Accordingly, a transcriptomal analysis was performed on brain tissue at 0, 7, 14, 21 and 28dpi from the HAT CD1/GVR35 mouse model. Up to 21dpi, most parasites are restricted to the blood and lymphatic system. Thereafter the trypanosomes enter the brain initiating the encephalitic stage. Analysis of ten different time point *Comparison* pairings, revealed a dynamic transcriptome comprising four message populations. All 7dpi *Comparisons* had by far more differentially expressed genes compared to all others. Prior to invasion of the parenchyma, by 7dpi, ~2,000 genes were up-regulated, denoted **[7dpi↑]** in contrast to a down regulated population **[7dpi↓]** also numbering ~2,000. However, by 14dpi both patterns had returned to around the pre-infected levels. The third, **[28dpi↑]** featured over three hundred transcripts which had increased modestly up to14dpi, thereafter were significantly up-regulated and peaked at 28dpi. The fourth, a minor population, **[7dpi↑-28dpi↑]**, had similar elevated levels at 7dpi and 28dpi. KEGG and GO enrichment analysis predicted a diverse phenotype by 7dpi with changes to innate and adaptive immunity, a Type I interferon response, neurotransmission, synaptic plasticity, pleiotropic signalling, circadian activity and vascular permeability without disruption of the blood brain barrier. This key observation is consistent with recent rodent model neuroinvasion studies and clinical reports of Stage 1 HAT patients exhibiting CNS symptoms. Together, these findings challenge the strict Stage1/Stage2 phenotypic demarcation in HAT and show that that significant neurological, and immune changes can be detected prior to the onset of CNS disease.

## Author summary

Human African Trypanosomiasis, (sleeping sickness), transmitted by the endemic tsetse fly, remains a public health and economic blight for around 70 million inhabitants of sub-Saharan Africa. After a variable period in the haemo-lymphatic system (Stage 1), the trypanosomes cross the blood brain barrier and enter the CNS initiating the encephalitic (Stage 2) of the disease defined by the host’s strong immune response with attendant neuropsychiatric debilitating symptoms including sleeping sickness, the hallmark symptom. If not treated, death invariably follows. Our understanding of the contribution of the host’s genetics to these two neuropathogenic events is poorly understood. Using a mouse trypanosomiasis model spanning 28 days, we compared the gene expression profiles of trypanosome infected and control mice brains over several time points. By day 7, the activity of over 4,000 genes were altered and genetic programmes, responsible for the development of the Stage 2 encephalitic symptoms were activated prior to any significant breakdown of the blood brain barrier. These findings challenge this traditional Stage1/Stage 2 phenotypic demarcation and accords with reports of Stage 1 patients presenting with Stage 2 encephalitic symptoms and the diagnosis of asymptomatic patients reflects the complex interaction between host and parasite genetics that impact on disease progression.

## Introduction

At least 70 million people in 36 countries throughout sub-Saharan Africa are at risk from the neglected tropical disease human African trypanosomiasis (HAT) commonly termed sleeping sickness. [1]. The condition results from infection of the morphologically indistinguishable extracellular haemoflagellate protozoan parasites, the West African form *Trypanosoma brucei gambiense* and the East Africa type *T*. *brucei rhodesiense* transmitted by the bite of the teste fly (*Glossina* sp). The disease progresses through two distinct clinical stages [2]. During the haemolymphatic Stage1, the trypanosomes invade and replicate in the blood, lymphatics and peripheral organs leading to a systemic inflammation with a variety of non-specific clinical symptoms. After a variable period, which is more prolonged in the *gambiense* form, trypanosomes invade the CNS initiating the encephalitic Stage 2 of the disease characterised clinically by protean neurological features and pathologically by the presence in the brain of trypanosomes, macrophages, lymphocytes, cytokines and chemokines in the CSF [3] and activation of microglia and astrocytes [4]. This exacerbates the cytokine mediated blood brain barrier (BBB) breakdown thereby amplifying the inflammatory response with a worsening debilitating neuropsychiatric impairment and the disruption of circadian rhythm control giving rise to major alterations of sleep structure, the hallmark CNS presentation [5]. If not diagnosed accurately, which is essentially reliant on CSF analysis due to the lack of robust clinical staging biomarkers, sleeping sickness is invariably fatal. As clinical investigation studies are severely hampered for numerous logistical reasons including ethical considerations and the very limited availability of post-mortem material, unravelling the neuropathogenesis of HAT has been and remains essentially dependent on a range of animal models, particularly the rodent paradigm [6, 7]. The well characterized human clinical phenotype is readily recapitulated in a range of rodent models infected with variety of trypanosome stabilates including the human resistant species *T*. *b*. *brucei*. The model employing the CD1 outbred mouse strain infected with the *T*. *b*. *brucei* GVR35 stabilate, has been widely used for over 30 years in molecular pathogenesis studies and provides the focus of the current investigation [8, 9]. In these various studies, the consensus has been that up to 21dpi, most parasites are essentially restricted to the haemolymphatic system, beyond which, the infection can no longer be effectively treated with Stage-1 drugs. If not then treated with Stage-2 drugs, the mice succumb to a combination of neurological and immunosuppressive complications resulting in death.

As recently reviewed [10], it had been thought that parenchymal invasion was preceded by the initial infection of the choroid plexus and the circumventricular organs *via* the fenestrated endothelial layer of the blood vessels in these regions. However, a series of experiments has challenged this strict Stage1/Stage2 demarcation. Fluorescently labelled *T*. *b*. *brucei* and *T*.*b*. *rhodesiense* parasites were detected in the parenchyma just a few hours post-infection [11] while in a rat model study [12] parasites and T cells were observed in the parenchyma at 9dpi. An updated re-examination of the CD1/GVR35 model was undertaken using qPCR, Contrast Enhanced Magnetic Resonance Imaging (CE-MRI) and histopathology. The study confirmed the presence of trypanosome DNA in brain homogenate at 7dpi, and cellular neuropathology and a significantly amplified CE-MRI signal at 14dpi, each criterion increasing incrementally at later time points [13].

These rodent model findings in conjunction with reports of CNS symptoms in Stage1 *rhodesiense* HAT patients suggest that BBB impairment may be more progressive and begin earlier than had been previously envisaged [14]. However, it should be emphasized that when and by what route(s) trypanosomes invade the CNS remains an ongoing and contentious issue. In fact, the presence of parasites in the parenchyma has been challenged with the suggested alternative of a restricted location in the pial space of the meninges following breakdown of the blood-CSF barrier [15] although at a later stage of infection, the trypanosomes may enter the glia limitans leading to encephalitis and death. Despite the wealth of genetic information accrued from targeted gene studies our understanding of the molecular mechanisms of the two critical events in HAT neuropathogenesis, the host’s dysregulated immune response and parenchymal invasion are poorly understood.

As reviewed [3, 16–18], the products of the *Tlr*9, *Tlr*2 and *Myd*88 genes have been shown to be critical determinants in the initiation of the innate response *via* activation the NFκB complex and subsequent expression of genes encoding the inflammatory cytokine mediators TNF-α, IFN α/β and IFN-γ which in turn induces the expression of a panel of the chemokine genes most notably *Cxcl*9, *Cxcl*10 *Cxcr*3 and *Ccl*5 the encoded products of which are integral to trans-endothelial leucocyte migration. This pro-inflammatory assault is counter balanced by the activity of several Th-2 anti-inflammatory molecules most notably IL6, IL10 and TGFβ-1and an ensuing immune suppression phenotype with devastating neurological consequences.

Due to the inherent structural and interconnected complexities of the endothelial neurovascular unit (NVU) [19], the adult quiescent BBB, though highly regulated is also fragile and susceptible to a range of insults leading to a variable degree of vascular leakage [20]. Despite an early dye study reporting BBB damage [21], to date, there is no convincing evidence of any permanent structural disruption of the paracellular complex of the BBB to facilitate trypanosome migration in rodent models [22, 23]. Other proteins mechanistically involved in trypanosome migration in rodent models are laminins 4 and 5 [24] and a small cohort of ECM metalloproteinases [25]. A leucocyte–trypanosome coupled neuroinvasion model mediated by the establishment of an IFNγ induced CXCL10 gradient in the astroglial endfeet of the NVU has been proposed [26] while reduced migration was observed for both trypanosomes and T cells into the parenchyma of TNF-α and IFN α/β null mice [18]. Treatment of cultured endothelial cells with the trypanosome encoded cysteine protease brucipain can challenge the molecular integrity of the parenchymal basement membrane resulting in transient perturbation of calcium signalling [27, 28].

The undoubted success of targeted studies has expanded our knowledge of the molecular genetics underpinning the HAT clinical phenotype including the assembly of the *African trypanosomiasis* ID^5143^ KEGG pathway (S1 Fig) [29] and its 38 participating genes. However, the full complement of differentially expressed genes that drive phenotypic change await identification. Notwithstanding well documented limitations, a microarray approach offers an unbiased experimental stratagem to monitor simultaneous differential gene expression during disease progression. Somewhat surprisingly, microprofiling of *T*.*b*.*brucei* infected CNS material has been restricted hitherto to two investigations by Amin and colleagues [26, 30] using the C57BL/6 inbred mouse infected with the *T*.*b brucei* stabilate AnTat 1.1E utilizing an older glass slide based microarray technology. The first [26], was essentially a targeted study on the expression of a panel of chemokine genes between the two pairings (6-15dpi) and (6-28dpi). The latter study was more global in approach [30], quantifying the total number of differentially expressed genes with a GO annotation from the three temporal pairings (6-15dpi), (6-28dpi) and (15-28dpi).

We report here the outcome of a microarray transcriptome analysis performed on whole brain tissue collected at 0, 7, 14, 21 and 28dpi from the CD1/GVR35 HAT mouse model using the commercial Illumina MouseWG6_V2_R3-11278593_A chip. Over 4000 differentially expressed genes were subjected to a combined KEGG pathway and GO term functional enrichment analysis. In addition, the transcriptome database was interrogated to assess the expression profile over the 28day timeline of documented and candidate trypanosomiasis genes. We surmise that by whatever neuroinvasion route(s) and mechanism(s) used, the presence of trypanosomes, wherever they reside in the CNS compartment, elicits changes in the host’s transcriptome providing genetic correlates with clinical phenotypic traits. We hypothesise here that the recent findings [11–13] of neurological features and parasite brain infiltration occurring at much earlier stages in HAT rodent models than previously thought might be explained by early activation of host genetic programmes controlling CNS disease. Our data shown below suggest that this is indeed the case.

## Materials and methods

### Ethics statement

All animal procedures were authorised under the Animals (Scientific Procedures) Act 1986, Home Office Licence PPL60/4414 and approved by the University of Glasgow Ethical Review Committee

### Mice and infections

Innocula of 2 x 10^4^ of *T*.*b*.*brucei* of the cloned stabilate GVR35 were intraperitoneally injected into adult female CD1 mice (Charles River Laboratories). Infected CD1/GVR35 mice develop a fluctuating parasitaemia and by 21dpi the mice cannot be treated with Stage 1 drugs. Mice chosen for this study were all healthy littermates of similar age and weight. Three replicate infected mice groups were euthanized and perfused at 7, 14, 21 and 28dpi in addition to a group of four non-infected control mice equivalent to 0dpi as previously described [8]. At 7dpi the animals were screened for the presence of trypanosomes by examination of wet blood films. All mice were found to be parasitaemic.

### Microarray analysis

Excised brains weighing around 500mg were immediately homogenized in 5ml RNABee (amsbio) and stored in 1ml aliquots at 80°C for subsequent microarray and end-point RT.PCR analyses. Total cellular RNA was prepared from infected and control brain (0dpi) replicates according to manufacturer’s guidelines. Briefly, the RNA samples were DNase treated (Ambion) and column purified using a RNeasy MiniEluate Kit (Qiagen). The integrity of the samples was assessed on an Agilent 2100 Bioanalyzer which provides an entire electrophoretic trace of the RNA sample that evaluates the extent of degraded ribosomal species expressed as a RIN value between 0 and 10. Multiple RNA preps were performed for each sample to ensure (3 x 7dpi), (3 x 14dpi), (3 x 21dpi), (3 x 28dpi) and (4 x non-infected controls (0dpi)) with RIN values ≥ 8.5 were available for custom microarray analysis outsourced to FIOS (Fios Genomics UK). Using the Ambion Illumina Total RNA Amplification Kit, the RNA samples were converted into cDNAs and subjected to T7 *in vitro* synthesis to generate biotinylated cRNA and hybridized to 50 oligomer probes printed on the MouseWG6_V2_0_R3_11278593_A BeadChip and stained with streptavidin–Cy3. A single WG6 Chip contains six whole genome microarrays each encoding 45,281 probes representing the 22,000 genes or so that comprise the mouse genome as estimated by UniProt as from August 2020. Raw data were transformed using a variance stabilizing transformation (VST) method prior to normalisation across all arrays using the robust spline normalisation (RSN) method. Relative transcriptional activity was converted to log_2_ expression units. From the full complement of the 45,281probeset, 23,213 were detected at least once across the arrays, with an expression hybridization signal intensity above the fluorescence background corresponding to a log_2_value ≥ 6.3, were retained for subsequent differential gene expression analysis.

Sixteen arrays to accommodate (3 x 7dpi), (3 x 14dpi), (3 x 21dpi), (3 x 28dpi) and (4 x 0dpi) samples underwent a QC check using the QualityMetrics Bioconductor package **[31]**. Arrays were scored using the four parameters, maplot, boxplot, heatmap and manual inspection where 15 arrays passed QC and a non-infected control (0dpi) failed. A total of 10 single and/or multi-factor *Comparisons* were analysed using linear modelling. Subsequently, empirical Bayesian analysis was applied using the Bioconductor package limma [32] and corrected for multiple testing using the Benjamini and Hochberg method [33]. For each *Comparison*, the null hypothesis was that there was no difference between the groups being compared. *Comparisons* were examined to identify expression differences at a strict statistical level (adjusted p-value <0.001). Based on this stringent threshold, significant probes in each Comparison were assessed using a hypergeometric test for functional enrichment (p<0.05) of KEGG pathways and GO terms (p<0.05) based on their annotation information.

### End-point RT.PCR analysis

End-point RT.PCR was performed on the same source of total cellular RNA samples that were earmarked for microarray experimentation. Reactions were carried out in the linear amplification range and message levels corresponding to the genes of interest were expressed relative to the activity of the mouse housekeeping gene *Cyclophilin*. In brief, and as detailed elsewhere [34], total cellular RNA was converted to cDNA using SuperScript III (Thermo Fisher Scientific-UK). PCR reactions were performed on 5ng of cDNA using RedTaq Ready Mix (Sigma-UK) against gene of interest primer sets. Thermal cycling parameters were, an initial denaturation step of 94°C/5 mins, a core cycle comprising (94°C/1 min)/ (55°C-65°C/1 min)/ (72°C/1 min) for 25–35 cycles was followed by a final extension of (72°C/10 mins. PCR products were separated by TAE gel electrophoresis, visualised by ethidium bromide staining and quantified by densitometry using an UVIdocD55XD documentation system (Uvitec UK).

## Results

### Generation and organization of raw data

**Fig 1:**
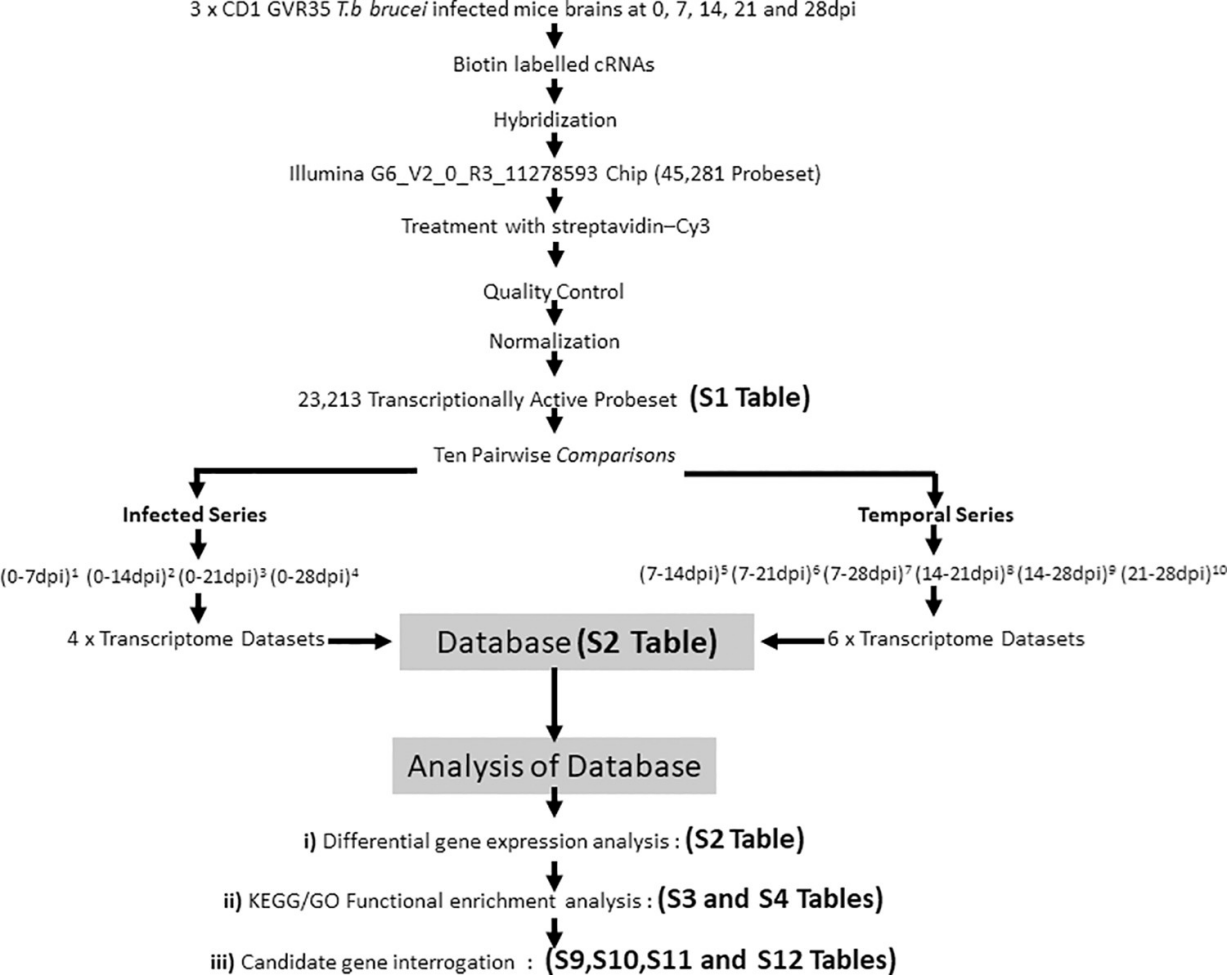
Experimental workflow chart depicting generation and organization of raw data and analysis of the transcriptome database. A microarray analysis was carried out on the brains of *T*. *b*. *brucei* infected mice collected at 0, 7, 14, 21 and 28dpi. Corresponding biotinylated cRNAs were hybridized to the Illumina G6_V2_0_R3_11278593 Chip encoding 45,281 oligonucleotide probes and treated with Streptavidin- generating a normalized 23,213 transcriptionally active probeset. Ten *Comparisons* distributed between the Infected Series [(0-7dpi)^1^ (0-14dpi)^2^ (0-21dpi)^3^ (0-28dpi)^4^] and the Temporal Series [(7-14dpi)^5^ (7-21dpi)^6^ (7-28dpi)^7^ (14-21dpi)^8^ (14-28dpi)^9^ (21-28dpi)^10^] generated ten transcriptome datasets which were subjected to three levels of analysis *viz* **i)** Differential gene expression analysis, **ii)** KEGG/GO Functional enrichment analysis and **iii)** Candidate gene interrogation.

As laid out in the schematic Workflow Chart (Fig 1), a microarray analysis using the Illumina MouseWG6_V2_0_R3_11278593_A Bead Chip was performed on GVR35 *T*.*b brucei* infected CD1 mouse brain RNAs collected in triplicate at 0, 7, 14, 21 and 28dpi. Biotin labelled cRNA was hybridized to the Bead Chip and probed with streptavidin-Cy3. After a series of quality control checks, only probes detected at least once across the arrays were retained for subsequent analysis. Following normalization to minimise variation gene expression measurement, 23,213 probes were designated as transcriptionally active, corresponding to over 11,000 genes. This figure represents a brain message complexity of around 50% similar to that reported from other mouse brain microarray studies but significantly less than the true mRNA complexity considered to be around 80% [35]. This disparity arises in the sensitivity of microarray detection as many CNS genes are expressed at low levels and the use of whole brain homogenate as the source of mRNA can masquerade regional brain expression differences. S1 Table lists this collection of normalized transcripts presented in alphabetical order alongside the log_2_ expression unit value for each of the four time points for each replicate group and the three non-infected control samples equivalent to 0dpi. Ten pair-wise *Comparisons* were performed on this active probeset. A *Comparison* nomenclature that specifies the two different time point combinations was adopted. The Infected Series compared the non-infected control equivalent at 0dpi against the four infected time points denoted (0-7dpi)^1^, (0-14dpi)^2^, (0-21dpi)^3^ and (0-28dpi)^4^. The Temporal Series measured activity changes between six different infected time point combinations *viz* (7-14dpi)^5^, (7-21dpi)^6^, (7-28dpi)^7^, (14-21dpi)^8^, (14-28dpi)^9^ and (21-28dpi)^10^. Expression difference for all *Comparisons* was set at a strict statistically significant adjusted p value <0.001 with an attendant adaptive threshold fold change with associated significance statistics.

The resultant transcriptome database (S2 Table) comprises a dataset for each of the ten *Comparison* is set out in order of decreasing adjusted p values and corresponding fold changes were subjected to **i)** differential gene expression analysis, **ii)** KEGG (S3 Table) and GO (S4 Table) functional enrichment analysis and **iii)** the database (S2 Table) was interrogated to assess expression profiles of candidate trypanosomiasis genes (S9–S12 Tables)

### Differential gene expression analysis

#### Quantitative transcriptome analysis

**Fig 2:**
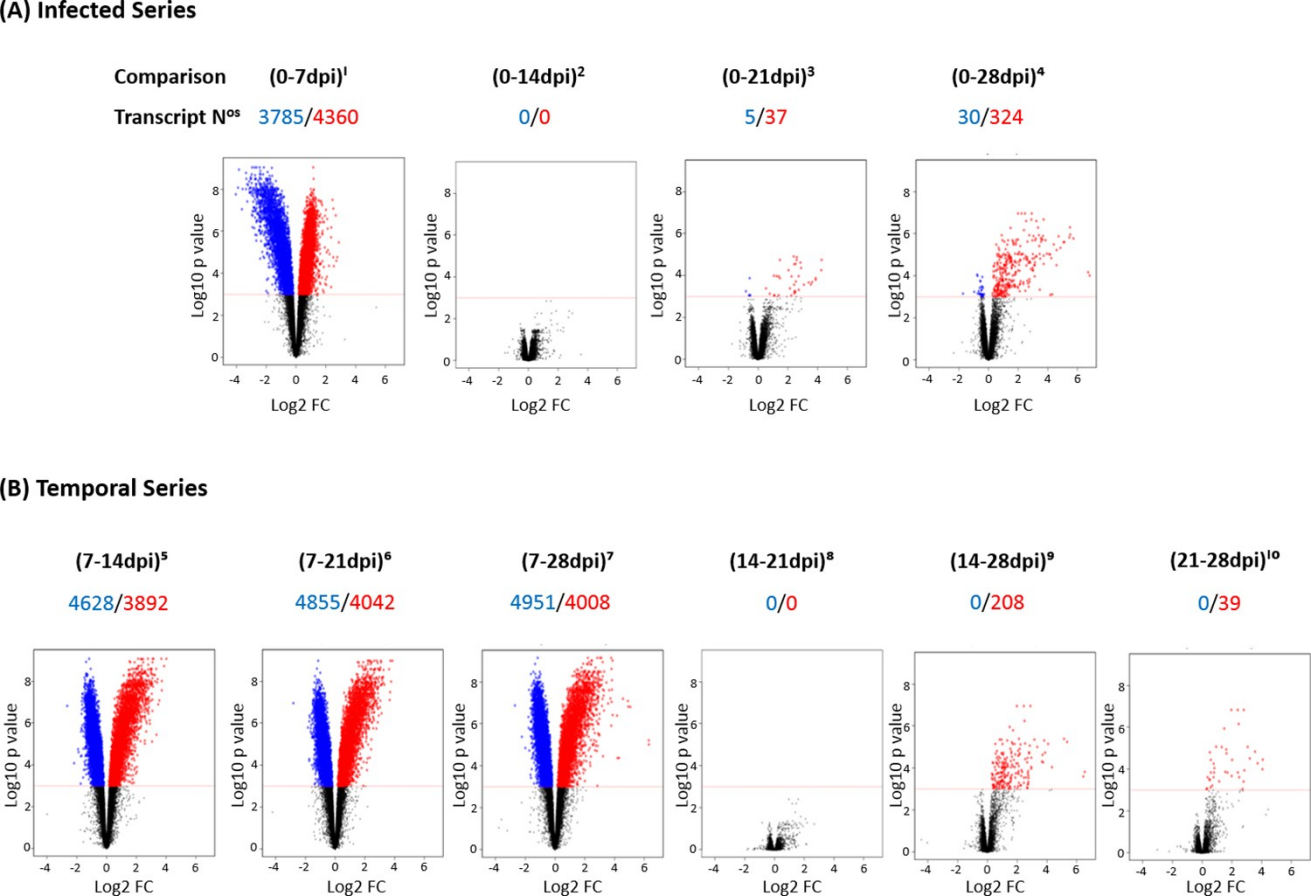
Volcano Plots with attendant up and down regulated transcript numbers for each *Comparison*. Volcano plots that chart statistical significance (p value) versus magnitude of change (fold change) on the y and x axes, respectively was performed on the brains of *T*. *b*. *brucei* infected mice at 0, 7,14, 21 and 28dpi on ten *Comparisons* comprising the Infected Series (A) and the Temporal Series (B). The number of up-regulated and down-regulated transcripts depicted in red and blue, are aligned below each the ten *Comparison* and corresponding volcano plot.

Inspection of the volcano plots (Fig 2A and 2B) which chart differential gene expression against fold change, with the numbers of up-regulated transcripts depicted in red and down-regulated samples in blue, reveal a dynamic quantitative transcriptome over the 28 day timeline unveiling two key observations. First, the 7dpi *Comparisons* in both Infected (Fig 2A) and Temporal Series (Fig 2B) had the greatest activity differences relative to all other *Comparisons*. Secondly, there was no significant differential gene expression in the (0-14dpi)^2^ and (14-21dpi)^8^ pairings. Transcript data for the Infection *Comparison* (0-7dpi)^1^ (Fig 2A) confirmed that by 7dpi the levels of 8145 mRNAs of the normalized probeset of 23217 were altered approximating to around 4000 genes. 4360 transcripts had increased and 3785 were down-regulated. The absence of any significant differential gene expression activity in the (0-14dpi)^2^ *Comparison* suggests that by 7dpi or earlier, one message population was up-regulated while the activity of the other had decreased, but by 14dpi or before, mRNA levels of both populations had readjusted to approximately those of the non-infected control (0dpi). This clearly demonstrates that the interval between 0 and 14dpi marks a period of intense transcriptional activity. However, it should be recognized that the host’s response may in part, be a consequence of a transitory systemic response to the initial trypanosome infection. Message activity levels in the (0-21dpi)^3^ and (0-28dpi)^4^ pairings with corresponding values of (37/5) and (324/30) are indicative of a post-14dpi period of increased transcription (Fig 2A). This cyclical activity between 0 and 14dpi followed by a post-14dpi induction was also evident in the Temporal Series profiles (Fig 2B).

A fixed FC, usually around ±1.5–2.0 is a consensual measure of significant differential gene expression activity in microarray analyses. S5 Table depicts transcript numbers (p<0.001) and non-statistically filtered ± FC values ranging from ± ≥2 to ≥32 for each *Comparison*. The highest FCs *viz* from ≥8 to ≥32 was recorded in the (0-28dpi)^4^, (7-28dpi)^7^ and (14-28dpi)^9^ pairings. The high FC values in (14-28dpi)^9^ provides supplemental evidence of post-14dpi induced population. The highest down-FC values *viz* ≥4 (295↓) and ≥8 (21↓) was essentially restricted to (0-7dpi)^1^.

When total transcript numbers were plotted against FC ≥ 2 (Table 1), only around 30% of the total transcript population of the 7dpi pairings (highlighted in shade), had a ± FC ≥ 2 compared to the higher values of the post-14dpi *Comparisons* such as 65.86% for (14-28dpi)^9^. This infers that transcriptional activity between 0 and 14dpi is less expansive when measured by FC. This besots high-throughput transcriptome studies as to the weighting to be given between p values and corresponding FC as the true index of biological significance. This was highlighted in a zebrafish heart tissue microarray study where the application of different FC reported different hypoxia outcomes [36]. Accordingly, we adopted a strict statistical significance of an adjusted p value of <0.001 and an adaptive FC threshold with its own associated significance statistics. This implies that although around 60% have a FC < 2.0 in (0-7dpi)^1^ their activity may well be biologically significant.

**Table 1:** Total transcript population plotted against the number of statistically significant mRNAs with ≥ 2 FC reveals variation in the level of gene activity when measured by p value differential gene expression and ≥ 2FC.

Comparison^#^	Total Transcript N^os^	≥ 2 FC	% ≥ 2 FC
**(0-7dpi)** ^**1**^	8145	2370	29.06%
**(7-14dpi)** ^**5**^	8520	2542	29.83$
**(7-21dpi)** ^**6**^	8897	2859	32.13%
**(7-28dpi)** ^**7**^	8959	2918	32.57%
**(0-14dpi)** ^**2**^	0	0	0%
**(14-21dpi)** ^**8**^	0	0	0%
**(0-21dpi)** ^**3**^	42	35	83.33%
**(0-28dpi)** ^**4**^	354	211	59.60%
**(14-28dpi)** ^**9**^	208	137	65.86%
**(21-28dpi)** ^**10**^	39	24	61.53%

#### Qualitative transcriptome analysis

*Comparisons* (0-7dpi)^1^, (7-14dpi)^5^, (0-28dpi)^4^ and (14-28dpi)^9^ that capture key significant quantitative transcriptome changes over the 28 day timeline (Fig 2) were selected for a limited qualitative analysis of the statistically most significant 20 genes for each *Comparison* (S5 Table) and of the 25 transcripts with the highest FCs across all *Comparisons* (Table 2).

**Table 2:** Twenty five genes with the highest ± FCs across all *Comparisons*.

Gene	Symbol	FC ↓^#^	Adj p-value	Gene	Symbol	FC ↑^#^	Adj p-value
Small nucleolar RNAgene 1	*Snhg*11	-16.46^1^	1.77E-08	CD74 antigen (invariant MHCII)	*Cd*74	44.94^4^	5.03E-07
Coatomer protein γ2	*Copg*2os2	-14.18^1^	1.18E-09	Cysteine and glycine-rich protein 1	*Csrp*1	41.76^1^	4.26E-03
Glutamate receptor α2	*Gria*2	-13.68^7^	7.30E-10	Chemokine (C-X-C motif) ligand 9	*Cxcl*9	34.58^4^	2.54E-06
Maternally expressed 3	*Meg*3	-12.02^1^	9.83E-09	Histocompatibility 2, class II A, β1	*H*2-*Ab*1	28.80^4^	4.72E-06
Proteolipid protein 1	*Plp*1	-10.76^6^	8.10E-09	Interferon gamma induced GTPase	*Igtp*	26.46^4^	1.34E-06
Zinc finger, RAN-binding 2	*Zranb*2	-10.05^1^	3.28E-09	Serum amyloid A 3	*Saa*3	24.13^4^	6.04E-06
Solute carrier family 24r2	*Slc*24a2	-9.71^1^	5.79E-09	Guanylate binding protein 2b	*Gbp*2	23.99^4^	3.16E-05
Eukaryotic initiation factor 5	*Eif*5	-8.98^5^	4.15E-07	Chemokine 5	*Ccl*5	21.13^4^	3.25E-06
Solute carrier family 17 6	*Slc*17a6	-8.85^1^	3.32E-09	Chemokine 13	*Cxcl*13	17.83^4^	9.86E-06
BCL2-transcription factor 1	*Bclaf*1	-8.52^1^	1.49E-08	Immunity-related GTPase	*Irgm*2	16.89^4^	9.44E-06
Kinesin family member 5C	*Kif*5c	-8.43^1^	8.47E-09	Coatomer protein subunit γ2	*Copg*2os2	16.21^6^	8.62E-10
Selenoprotein T	*Selt*	-8.29^1^	1.39E-08	Lysozyme 1	*Lyz*1	13.14^4^	2.14E-07
Ca/calmodulin kinase IV	*Camk*4	-8.23^1^	8.47E-09	Histocompatibility 2, K locus 2	*H*2-*K*2	12.72^4^	1.30E-06
Gametogenetin binding	*Ggnbp*2	-7.87^1^	1.97E-09	Predicted gene 8909	*Gm*8909	12.54^4^	1.92E-06
Unc-13 homolog C	*Unc*13c	-7.76^1^	1.91E-08	CD52 antigen	*Cd*52	11.84^4^	1.85E-06
LUC7-like 3 (S. cerevisiae)	*Luc*7l13	-7.74^1^	9.24E-10	Serine peptidase inhibitor, A, 3G	*Serpina*3g	11.66^4^	2.41E-06
Diacylglycerol kinase, beta	*Dgkb*	-6.50^1^	5.79E-09	Lysozyme 2	*Lyz*2	11.65^4^	1.64E-06
Muscleb splicing factor	*Mbnl*1	-6.251	9.22E-09	Proteasome β type 8 peptidase 2	*Psmb*8	11.19	1.56E-05
Myosin VA	*Myo*5a	-5.91^1^	1.04E-08	Interferon regulatory factor 1	*Irf*1	10.90	9.28E-06
Microfibrillar protein3	*Mfap*3l	-5.62^1^	2.34E-07	Complement component 3	*Gbp*3	10.54	1.94E-06
Ankyrin repeat domain 12	*Ankrd*12	-5.59^1^	9.42E-07	Chemokine (C-X-C motif) ligand 10	*Irgm*1	9.39	1.21E-05
Dynamin 1-like	*Dnm*1l	-5.44^1^	9.24E-10	Lymphocyte Ag 6 complex, locus A	*Ly*6a	9.22	1.21E-05
SMG1 homolog	*Smg*1	-5.41^1^	1.22E-07	Complement component 3	*C*3	8.83	2.07E-06
Family sequence 49B	*Fam*49b	-5.29^1^	1.02E-07	Fc receptor, IgG, low affinity IV	*Fcgr*4	8.76	2.32E-05
Janus kinase microtubule 2	*Jakmip*2	-5.18^1^	3.53E-07	Chemokine (C-X-C motif) ligand 10	*Cxcl*10	8.72	3.56E-05

FC ↓^#^ Down-regulated genes with corresponding Comparison number^#^

FC ↑^#^ Up-regulated genes with corresponding Comparison number^#^

FC ↓^#^ Down-regulated genes with corresponding Comparison number^#^

FC ↑^#^ Up-regulated genes with corresponding Comparison number^#^

As depicted in shade (S5 Table), 15 of the 20 (0-7dpi)^1^ down-regulated genes were up-regulated in the (7-14dpi)^5^ *Comparison*. The shared (0-7dpi)^1^ and (7-14dpi)^5^ qualitative profiles, although phenotypically disparate, could be grouped into a range of functional categories including modulation of expression (*Luc*7l3, *Prpf*18, *Pdcd*4, and *Gpbp*1), neuronal activity (*Gria*2, *Cdkl*2, *Ggnbp*2 and *Ccdc*174) pleiotropic signalling cascades (*Cacn*b4, *Ccpg* and *Pak*3) and mitochondrial activity (*Dnm*1l and *Mrpl*50).). This contrasts with the exclusive immune content of (0-28dpi)^4^ and (14-28dpi)^9^ sharing eight common genes shown in shade (S5 Table) comprises the innate response (*Saa*3), IFN induced Interferon Stimulated Genes (ISGs) (*Ifitm*1, *Ifitm*3, *Gm*4951and *Gbp*3), chemokines (*Cxcl*9, *Ccl*4, *Ccl*5, *Ccr*5 and *Cxcl*16), complement activity (*C*3), the adaptive response *Cd*3g including antigen presentation (*Cd*74, *H*2-*K*2 and *H*2-*Q*8), dysfunctional immunity (*Aif*1) and the bacteriolytic enzymatic activity (*Lyz*1 and *Lyz*2).

Examination of the 25 transcripts with the highest FCs across all *Comparisons* (Table 2) unveiled a similar demarcation between an early neuronal dominated phenotype and the post-14dpi immune dominated phenotype. All down-regulated genes were (0-7dpi)^1^ entries. This included changes to the gene regulatory machinery (*Zranb* and *Eif5*,), mitochondrial activity (*Dnm*1), signalling cascades (*Camk*4, *Dgkb* and *Mfap*3l), neurotransmission (*Gria*2, *Kif*5c, *Slc*17a6, *Selt* and *Unc*13c) and neuronal activity (*Slc*24a2 and *Myo*5a). The inclusion of the *Plp*1 gene (corroborated by end-point RT.PCR) is difficult to explain as it encodes the major protein of the CNS myelin sheath. In addition, the significance of the imprinting entries *Copg2os*2 and *Meg*3 is difficult to evaluate. Except for *Csrp*1 (neuronal development) and *Copg*2os2 (imprinting) all other up-regulated mRNAs were found in (0-28dpi)^4^ and immune exclusive. This featured the antigen presenting genes (*Cd*74, *H*2-*Ab*1and *H*2-*K*2). Additional functional groupings comprised complement genes (*C*3 and *C*4a), IFNγ ISGs genes (*Igtp* and *Gbp*2), the IFNα,β ISG (*Irf*1) and the chemokine genes (*Cxcl*9, *Cxcl*13 and *Ccl*5) in addition to *Cxcl*10 which has been strongly implicated in trypanosome invasion [26]. Increased levels of *Fcgr*4 mRNA encoding a Fc gamma receptor is indicative of IgG activity.

#### Temporal gene expression patterns

**Fig 3:**
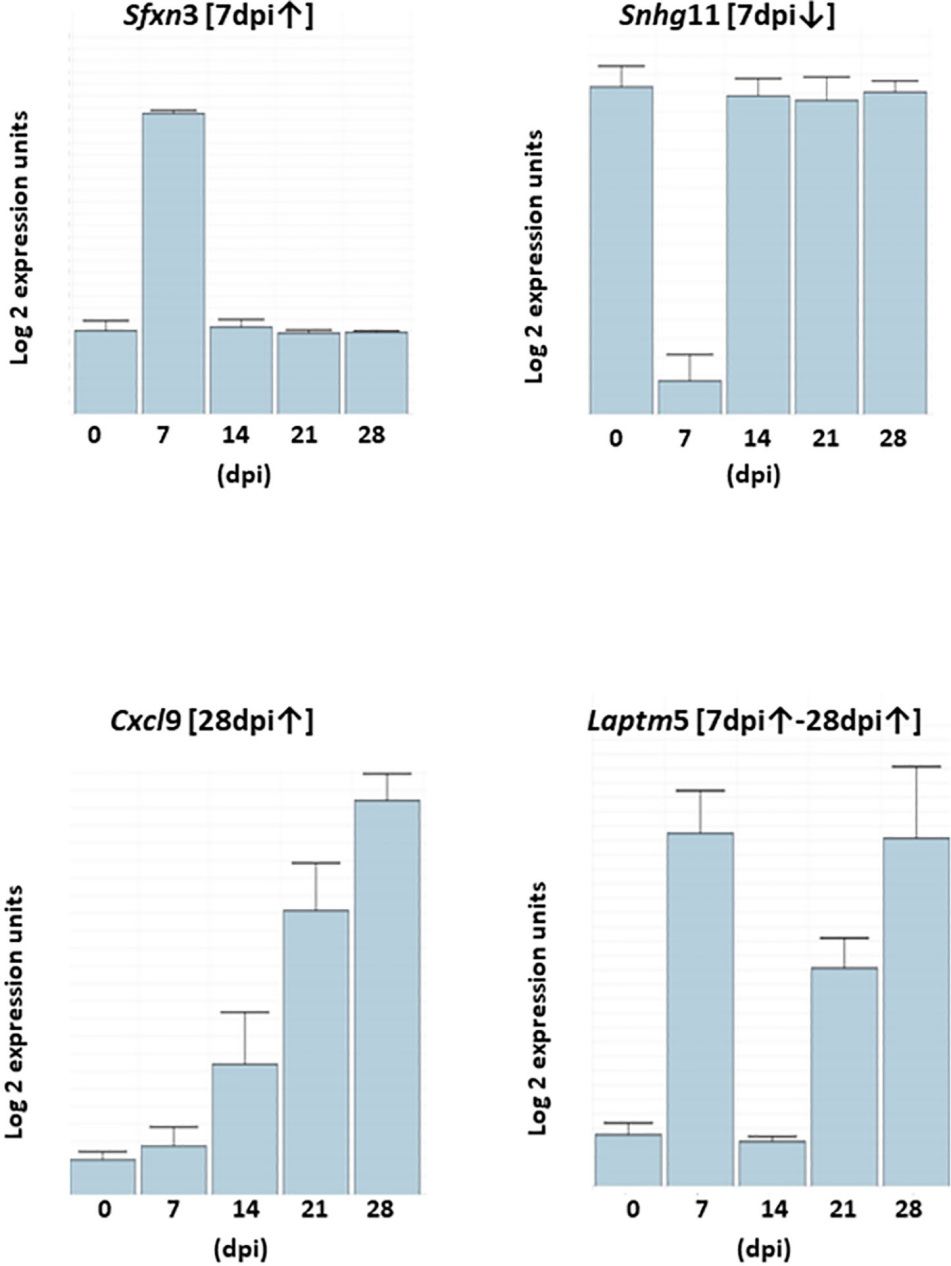
Representative genes for each of the four temporal generic gene expression patterns. The majority of differentially expressed genes could be assigned to one of four temporal generic expression patterns over the 28 day timeline designated **[7dpi↑]** *eg* (*Sfxn*3), **[7dpi↓]** *eg Snhg*11, **[28dpi↑]** *eg Cxcl*9 and **[7dpi↑-28dpi↑]** *eg* (|*Laptm*5). The two 7dpi patterns were by far the most prevalent describing the activity of over 8,000 transcripts between them while **[28dpi↑]** and **[7dpi↑-28dpi↑]** chart the activity of over 300 immune genes. In contrast to the representative genes, *Sfxn*3 and *Snhg*11 many mRNAs showed more gradation post-14dpi particularly the **[7dpi↑]** population. Some degree of post-14dpi gradation was also evident than the highly stylized patterns of *Cxcl*9 and *Laptm*5.

Most of the significantly active transcripts could be grouped into one of, or variations of, four broad generic temporal gene expression patterns denoted **[7dpi↑]**, *eg Snhg*11, **[7dpi↓]**, *eg Sfxn3*, (**[28dpi↑]**, *eg Cxcl*9) and **[7dpi↑-28dpi↑]**, *eg Laptm*5 (Fig 3). The first two, define the functionally disparate phenotype between 0 and 14dpi with respective peak and minimum expression at 7dpi. However, it should be appreciated that in many cases and in contrast to the highly stylized patterns represented by *Snhg*11 and *Sfxn3*, many examples showed more gradation in the message levels between 14dpi and 28dpi particularly in the **[7dpi↑]** population. The **[28dpi↑]** profile represented by the chemokine gene *Cxcl*9, is characterized by an initial and variable change in message levels between 0 and 7dpi followed by a distinct post 14dpi inducible period, typically with attendant high fold changes describes the immune dominated *Comparisons* (0-28dpi)^4^ and (14-28dpi)^9^ (Tables 2 and S5). The fourth, **[7dpi↑-28dpi↑]** represent mainly immune genes, have a characteristic twin peak pattern of similar levels as represented by the lysosomal-associated protein transmembrane 5 gene (*Laptm*5). As the profiles are generated from quantified message abundance at five fixed time points, they cannot measure the extent or otherwise of the temporal length of up-regulated plateaus and repressed troughs. For example, peak levels in **[7dpi↑]** could feasibly plateau from 3dpi to 8dpi or equally from 6dpi to 11dpi. Accordingly, these patterns should be viewed more as useful expression trends.

### Functional enrichment analysis

To gain insight of the phenotype predicted from the qualitative transcriptome database (S2 Table), a KEGG [29] and GO [37] functional enrichment analysis of all *Comparisons* was undertaken as detailed in S3 and S4 Tables respectively. Due to the GO annotation selection criteria, the number of GO terms far exceeds KEGG pathways entries (Table 3) confirming the dominant down-regulated pattern of (0-7dpi)^1^ followed by the subsequent up-regulation in (7-14dpi)^5^ and the post-14dpi induction in the (0-28dpi)^4^ and (14-28dpi)^9^ pairings.

**Table 3:** Quantification of up-and down-regulated KEGG pathways and GO terms for each *Comparison*.

	KEGG Pathways		GO Terms	
**Infection Series**	**Up**	**Down**	**Up**	**Down**
(0-7dpi)^1^	15	67	529	1047
(0-14dpi)^2^	0	0	0	0
(0-21dpi)^3^	10	0	286	11
(0-28dpi)^4^	50	1	1250	75
**Temporal Series**	**Up**	**Down**	**Up**	**Down**
(7-14dpi)^5^	70	18	1046	487
(7-21dpi)^6^	54	17	1050	584
(7-28dpi)^7^	80	21	1408	451
(14-21dpi)^8^	0	0	0	0
(14-28dpi)^9^	48	0	1020	0
(21-28dpi)^10^	0	0	10	0

**Fig 4:**
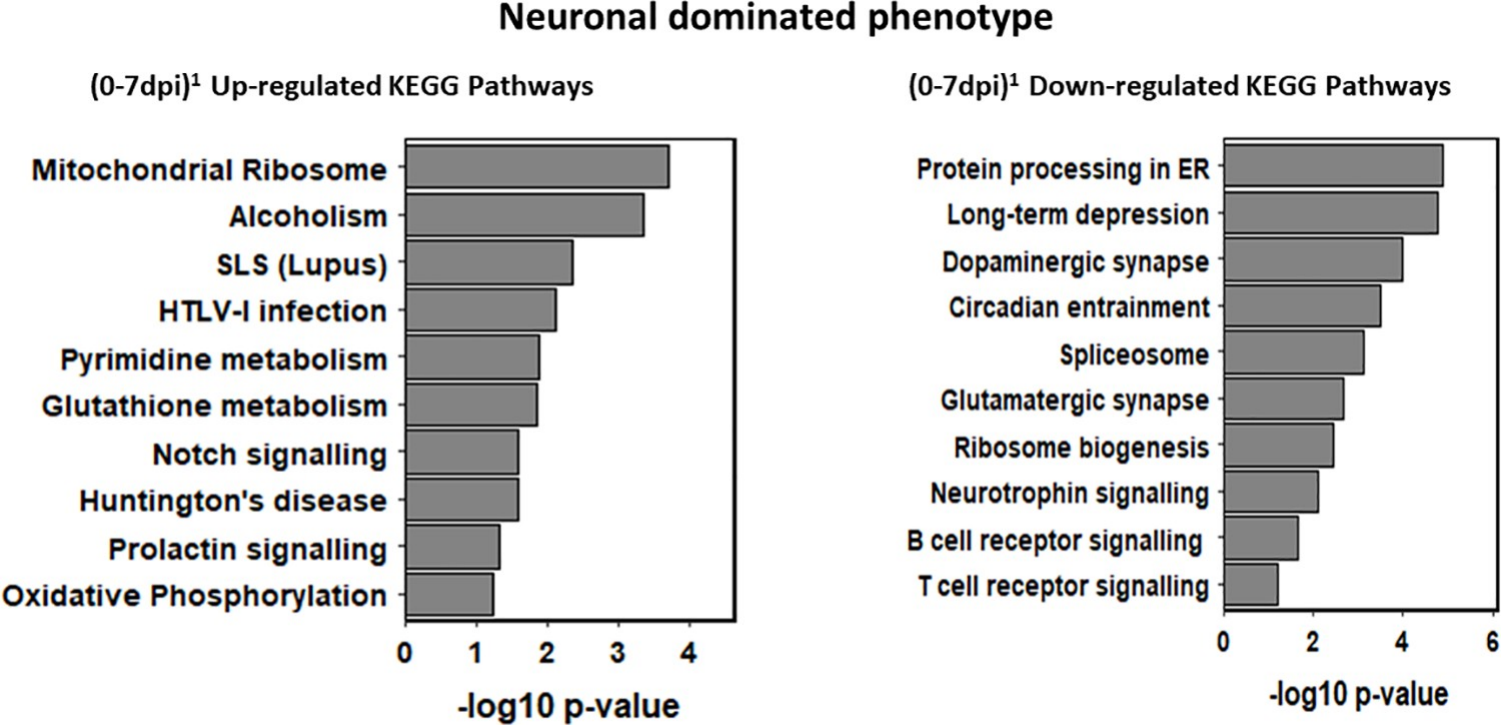
KEGG bar graphs for ten selected up and down-regulated KEGG pathways from the (0-7dpi)^1^ *Comparison* reveals an early neuronal dominated phenotype. Ten key up- and down-regulated pathways plotted against p values were selected to represent biological change in the (0-7dpi)^1^ *Comparison*.

**Fig 5:**
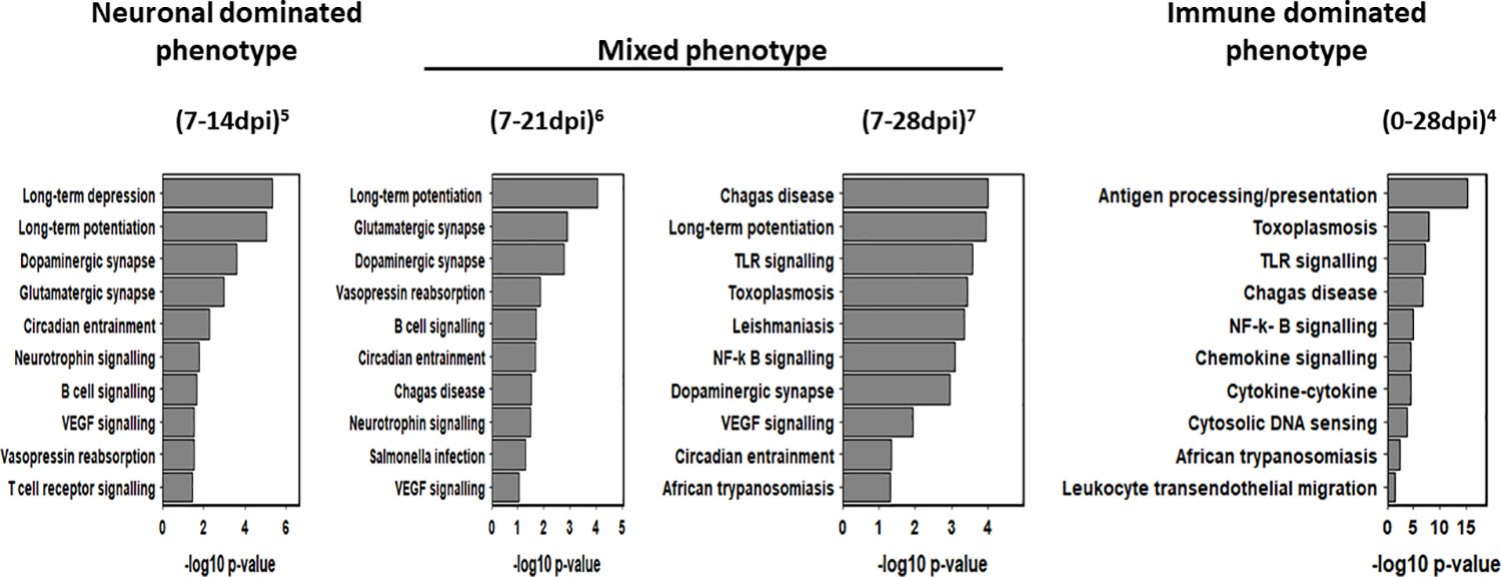
KEGG bar graph for ten up-regulated KEGG pathways selected from *Comparisons* (7-14dpi)^5^, (7-21dpi)^6^, (7-28dpi)^7^ and (0-28dpi)^4^ depicting an early neuronal dominated phenotype and its transition to an immune-dominated phenotype during disease progression. Ten key upregulated pathways plotted against p values were selected to represent biological change for each *Comparison*.

Enrichment profiles for all *Comparisons* collated in heatmaps (S2–S5 Figs) catalogue the statistically most significant 75 KEGG and GO entries. This confirmed two distinct phenotypes which are boxed in each heatmap between the functionally diverse neuronal dominated phenotype represented in (0-7dpi)^1^ and (7-14dpi)^5^ and the post-14dpi immune dominated *Comparisons* (0-28dpi)^4^ and (14-28dpi)^9^ share an almost identical up-regulated KEGG and GO heatmap fingerprint (S2 and S4 Figs). There was no significant KEGG or GO enrichment of down regulated genes outwith the 7dpi *Comparisons*. The S2 Fig pathways, *Oocyte meiosis* ID^4114^, *Prostate cancer* ID^5215^ and *Pancreatic cancer* ID^5212^ do not have a CNS phenotype. This, an inherent issue of enrichment analysis termed “crosstalk” by Donato [38] arises from common genes participating independently in different pathways and GO terms. A more detailed KEGG analysis supplemented when appropriate with GO data, was performed to compare the phenotypes between (0 and 14dpi) and (14 and 28dpi). KEGG pathway identifiers, sourced from S3 Table were grouped into a range of functional categories reflecting phenotypic heterogeneity over the 28 day timeline (S6–S8 Tables) are summarized in the KEGG bar graphs (Figs 4 and 5) plotting ten key pathways against p values for each *Comparison*.

#### Phenotypic analysis between 0 and 14dpi

As the up-regulated profile of (0-7dpi)^1^mirrors the contents of down-regulated pathways of (7-14dpi)^5^ and *vice versa*, KEGG analysis between 0 and 14dpi was restricted to the contents of (0-7dpi)^1^ (Fig 4 and S5 Table). The 12 up-regulated (0-7dpi)^1^ pathways (S6 Table) were classified into five broad functional categories. Selected pathways to illustrate this phenotypic diversity are listed in Fig 4 comprising mitochondrial activity (*Oxidative phosphorylation* ID^0190^), neuronal dysfunction (*Huntington* disease ID^5016^), metabolism (*Pyrimidine metabolism* ID^0240^), pleiotropic signalling (*Notch signalling* ID^4330^) and immune response (*Systemic lupus erythematosus* ID^5322^) in addition to GO evidence of an early immune response supported by *Interferon signalling* GO:^0060338^ and *Regulation of innate response* GO:^0002758^.

Forty of the 67 selected down-regulated (0-7dpi)^1^ KEGG identifiers sourced from S3 Table were categorised into 11 broad functional groupings (S6 Table). As depicted in Fig 4 this revealed a neuronal dominated phenotype characterised by a down-regulation in Transcription (*Ribosome biogenesis* ID^3008^) and Protein processing (*Protein processing in ER* ID^4141^) supported by an array of down-regulated GO terms including *RNA Regulation of Gene expression* GO:^0010468^ and *Translation initiation* GO:^0006413^. Disturbance of the neurotransmission network was predicted by the inclusion of eight pathways including *Serotonergic synapse* ID^4726^, and *Dopaminergic synapse* ID^4728^ in addition to modulation in the level of synaptic plasticity (*Long-term depression* ID^4730^ and *Long-term potentiation* ID^4720^. Although *Circadian Entrainment* ID^4713^ was down-regulated, the activity levels of the three GO terms, *Entrainment of circadian clock* GO:^0009649^, *Entrainment of circadian clock by photoperiod* GO:^0043153^ and *Photoperiodism* GO:^0009648^ had increased. Changes to a set of pleiotropic signal transduction pathways [39] integral to CNS homeostasis were recorded including *MAPK signalling* ID^4010^, *cAMP signaling* ID^4024^ and *ErbB signalling* ID^4012^ in addition to the neural specific pathway *Neurotrophin signalling* ID^4722^ and *Calcium signalling* ID^4020^ with critical roles in neurotransmission, excitability and long-term plasticity (S6 Table). There was a decrease in B and T cell signalling in addition to *Rig1-like receptor signalling* ID^4622^ and *Nod like receptor signalling* ID^4621^ involved in viral and bacterial pathogen pattern recognition respectively (S6 Table). In contrast five Type I interferon GO terms represented by *Type I interferon signalling pathway* GO:^0060337^ and two innate terms including *Regulation of innate immune response* GO:^0045088^ were up-regulated.

#### Phenotypic analysis between 14 and 28dpi

KEGG enrichment analysis of up-regulated genes of the Temporal *Comparisons* (7-14dpi)^5^, (7-21dpi)^6^ and (7-28dpi)^7^ identified 70, 54 and 80 pathways respectively. Twenty five were chosen to represent the range of biological change (S7 Table). The two Infected parings (0-21dpi)^3^ and (0-28dpi)^4^, yielded 12 and 50 pathways (S8 Table). As detailed in S7 Table the balance of entries in several of the functional categories between (7-14dpi)^5^ and (7-28dpi)^7^ were altered, most notably, those associated with neurotransmission, synaptic plasticity, pleiotropic signalling and the immune response, while pathway numbers describing BBB activity, apoptosis and circadian activity categories were equally represented in all three Temporal *Comparisons*. A summary of the content analysis of S7 and S8 Tables have been combined in Fig 5 depicting a distinct progression from a neuronal dominated phenotype defined in (7-14dpi)^5^ that increases in the immune content of the (7-21dpi)^6^ and (7-28dpi)^7^ mixed phenotypes culminating in the appearance of an immune dominated (0-28dpi)^4^ phenotype. Several (7-14dpi)^5^ neurotransmission and synaptic plasticity pathways including *Glutamatergic synapse* ID^4724^ and *Long-term potentiation* ID^4270^ active in (7-21dpi)^6^ and to lesser extent in (7-28dpi)^7^ marked the transition from a neuronal dominated phenotype (7-14dpi)^5^ to mixed neuronal–immune phenotype in (7-28dpi)^7^. Evidence for this transition in (7-21dpi)^6^ was the inclusion of four immune response categories including *Salmonella infection* ID^5132^ and *Chagas disease* ID^5142^ (Fig 5). Moreover, there was a significant increase in the immune content between (7-21dpi)^6^ and (7-28dpi)^7^ (Fig 5) evidenced by the four protozoan diseases, *African trypanosomiasis* ID^5143^, Chagas *Disease* ID^5142^, *Leishmaniasis* ID^5140^ and *Toxoplasmosis* ID^5154^ in addition to the innate markers *TLR signalling* ID^4620^ and *NF-k B signalling* ID^4064^.

Compared to the (7-21dpi)^6^ and (7-28dpi)^7^ mixed phenotypes (S7 Table and Fig 5), the (0-21dpi)^3^ 12 listed pathways listed in the 25 (0-28dpi)^4^ entries, were exclusively immune (S8 Table and Fig 5). As both (0-28dpi)^4^ and (14-28dpi)^9^ share 48 of 50 pathways (S3 Table), the 25 selected (0-28dpi)^4^ pathways were grouped into seven functional categories (S8 Table) Five of these, feature in Fig 5 including innate activity (*Cytosolic DNA sensing* ID^4623^, *TLR signalling* ID^5166^ and *NFkappaB* ID^4630^), adaptive activity (*Antigen processing and presentation* ID^4612^) immune-signalling (*Chemokine signalling* ID^4062^ and *Cytokine-cytokine receptor interaction* ID^4060^), protozoan infection (*Toxoplasmosis* ID^5145^, *Chagas Disease* ID^5142^ and *African trypanosomiasis* ID^5143^) and diapedesis (*Leukocyte trans-endothelial migration* ID^4670^). Further evidence of diapedesis genetic activity was the inclusion of *Cell Adhesion Molecule* ID^4514^ (S7 Table) corroborated by three up-regulated “leucocyte” and four “T cell” migration GO terms *eg Leucocyte migration* GO:^0050900^ and *Positive regulation of T cell migration* GO:^2000406^ in both (0-28dpi)^4^ and (14-28dpi)^9^ (S4A Table).

Despite CE-MRI evidence of an increase in barrier permeability in GVR35/CD1 brains [13], there was no significant change in the activity of the *Tight junction* ID^04530^ (S3 Table) or in any of the GO “tight junctions” or “blood brain barrier” GO terms (S4 Table). However, the up-regulated activities of *Blood vessel development* GO:^0001568^, *Vasculature development* GO:^0001944^ and *Blood microparticle* GO:^0072562^ terms in (0-28dpi)^4^ may be indicative of a physiological disturbance of vasculature homeostasis (S4A Table).

### Expression profiling of candidate genes

The transcriptome database (S2 Table) was interrogated to determine the 28 day expression patterns of the 38 *African trypanosomiasis* ID^5143^ genes and a panel of proposed Stage 2 biomarker genes (S9 Table) in addition to candidates associated with pathogenic change (S10 Table), the host’s immune response (S11 Table) and BBB integrity/impairment (S12 Table). Although default expression parameters were initially set to an adjusted p value of < 0.001 and FC around 1.5, the significance of these parameters was also assessed within the context of individual gene function and expression pattern. The format of these S Tables depicts the *Comparison* number with the maximum fold change denoted (Max FC^#^), its corresponding adjusted p value and best fit transcription expression pattern (Fig 3) for each gene.

#### African trypanosomiasis ID^5143^ and Biomarker genes

Of the 38 *African trypanosomiasis* ID^5143^ pathway genes (S9A Table) nine could not be detected including *Kng*1 and 2 encoding the kallikrein-kinin hormonal cascade in addition to *Il*12b and the E-selectin gene *Sele* while the levels *Il*12a, *Apo*a1, *Fasl* and *Plcb*2 message were subthreshold. The remainder were significantly active describing innate immunity (*Myd*88 and *Tlr*9), cytokine activity (*Ifnγ* and *Il1*β), diapedesis (*Vcam*1 and *Icam*1) globin synthesis (*Hba*-a1 and *Hbb*-b2), tryptophan metabolism (*Ido*1) and apoptosis (*Fas*) all defined by the **[28dpi↑]** or **[7dpi↑-28dpi↑]** expression patterns in contrast to *Thop*1 (circadian activity) and six neuroinvasion genes (*eg Gnaq* and *Prkc*a) that shared a **[7dpi↑]** profile. The expression profiles of a panel of candidate biomarkers [40–42] based on elevated Stage 2 CSF levels of CXCL8, CXCL10, CXCL13, ICAM1, VCAM1, beta-2-microglobulin (B2M), MMP-9, SSP1 (Osteopontin), the human fatty acid binding protein-3 (FABP) and the GTP metabolite, neopterin were assessed (S9B Table). The chemokines, *CAM*s and *B2m* mRNAs peaked at 28dpi with attendant high FCs. The *Gch*1gene encoding guanosine triphosphate cyclohydrolase responsible for neopterin synthesis was up-regulated by 7dpi while *Spp*1 encoding osteopontin a secreted phosphoprotein was repressed at 7dpi compromises their biomarker candidacy in this mouse model. The activities of *Fabp*3 and *Mnp*9 were subthreshold. Amin et al [30] identified 13 non-chemokine/cytokine secreted biomarkers six of which *B2m Grn*, *Reln*, *Tspo*, *Slpi*, and *Lcn*2 had comparable FCs. The extrahepatic gene *Saa*3, encoding the serum amyloid acute phase protein SAA3 which is secreted into the CSF had a classic **[28dpi↑]** pattern with a 26.4 FC may justify biomarker investigation.

#### Pathogenic genes

The levels of 8145 transcripts were altered by 7dpi (Fig 2). Screening this population identified 12 disparate functional categories (S10 Table) that may lead to pathogenic change. This listing comprised 11 entries involved in neurotransmission *eg* (*Kif*5c **[7dpi↓]**) three CAM genes *eg* (*Cntn*1 **[7dpi↓])**, 14 apoptosis gene bank members (ApoCanD) [43] *eg* (*Bicr*2 **[7dpi↓])** and eight *Apoptosis* ID^4210^ participants *eg* (*Pik*3r1 **[7dpi↓]**), a cohort of heat shock proteins *eg* (*Hspa*9 **[7dpi↓]**) which may have a role in neural proteostasis and neuroinflammation [44] and eight members of the *Dnaj*/*Hsp*40 gene family encoding co-chaperones for HSP70 proteins [45] *eg* (*Dnajc*4 **[7dpi↑]**). Fifty genes of the multi-functional Coiled-coil domain (*Ccdc*) family [46] were active *eg* (*Ccdc*124 **[7dpi↑]**). In addition, there was an up-regulation of five lysosomal protease storage cathepsin genes *eg* (*Cts*Z **[7dpi↑-28dpi↑]**). Four Serpin genes *eg* (*Serpina* 3g), belong to a large family of protease inhibitors [47] had a **[28dpi↑]** incremental pattern. There was a spread of core histone gene activity *eg* (*H2ac*7 **[7dpi]**). In addition, to their global role in the cell cycle regulation, histone core and histone modifying genes dominate the gene profiles of the immune disease pathway *SLS (Lupus)* ID^5322^ and the dysfunctional synaptic activity that defines *Alcoholism* ID^5034^ pathway.

The human *Slc* super-family with over 400 transporter genes organized into 65 functional families [48] have crucial neurologic roles including synaptic activity and in conjunction with the *Abc* gene family, regulate the efflux and influx of small molecules across the BBB. Changes to this expansive transport network can disrupt CNS homeostasis as documented by the 71 *Slc* genes associated with human brain disorders [49]. In the mouse, over 250 genes were expressed in the brain [50]. By 7dpi around 100 were up-regulated while the activity of over 50 were decreased by 7dpi (S2 Table). The 26 selected represent the breadth of CNS function (S10 Table) including nine members were associated with neurotransmission including *eg Slc*6a9 **[7dpi↑]** and *Slc*17a6 **[7dpi↓]**, six were enriched in the BBB [51] *eg Slc*2a13 **[7dpi↓]** and *Slc*1a3 **[7dpi↓]** encoding the glucose transporter GLUT13 and the glutamate transporter EATT respectively and six Circadian Gene Data Base (CGDB) genes [52]. In contrast to the vast majority of *Slc* genes which have a 7dpi expression pattern, *Slc*O2b1 **(**anion transport) *Slc*15a3 (proton-dependent transport of histidine) and *Slc*11a1 had a **[7dpi↑-28dpi↑]** profile.

A scan of the (0-7dpi)^1^ dataset identified around 50 circadian gene candidates, the majority, entries in the CGDB **[52]**. Included in the selected 26 (S10 Table) were a subset of SCN enriched core “clock” genes [53] was the eponymous *Clock* gene **[7dpi↓]** and *Per*1 **[7dpi↑]** both of which have been reported to be altered in rodent models [54, 55]. The remainder of candidates were classified as SCN enriched non-clock [56] including four high FC genes (S10 Table) with established roles in signalling or rhythmicity were the down regulated AMPA glutamate receptor genes *Gria*1 and *Gria*3, *Itpr*1 (Calcium signalling) and *Scg*2 encodes a member of the secretogranin family of neuroendocrine secretory proteins. An earlier study [57] reported a reduction in GRIA2 and GRIA3 levels in SCN slice preparations consistent with our finding on the reduced levels of the corresponding message levels in (0-7dpi)^1^. Other candidates of note included *Avp* **[7dpi↑]** and *Vip* **[7dpi↓]** encoding vasoactive peptides, crucial for regulated SCN output following photic stimulation, the *Pmch* **[7dpi↓]** and *Hcrt* **[7dpi↑]** genes encoding the contrasting sleep-promoting melanin and wake-promoting orexin neuropeptides respectively and the *African trypanosomiasis* ID^5143^ *Thop*1 gene **[7dpi↑]**. Given that most SCN neurons are GABAergic, the activities of two of the receptor genes *Gabr*b1 **[7dpi↓]** and *Gabr*d **[7dpi↑]** may be significant.

Changes occur in aromatic amino acid metabolism during trypanosomiasis including a decrease in tryptophan levels [58]. The activity of three metabolic tryptophan genes, *Ccbl*1 **[7dpi↑]**, *Inmt* **[7dpi↑]** and *Tph*2 **[7dpi↓]** was altered (S10 Table). Tryptophan levels, in part are regulated by indoleamine 2,3-dioxygenase, encoded by the *African trypanosomiasis* ID^5143^ *Ido*1gene (S1 Fig) was incrementally expressed **[28dpi↑]**. This is a rate limiting enzyme in the kynurenine pathway, several pathway catabolites are associated with the inflammatory response [59]. Despite the importance of the pro-inflammatory neuropeptide Substance P [60] in addition to prostaglandins D2 and E2 and nitric oxide as depicted in the *African trypanosomiasis* ID^5143^ no significant changes were detectable in any of the corresponding genes.

There is no documented evidence of a hypoxic episode during trypanosome infection. Despite subthreshold p values, four *African trypanosomiasis* ID^5143^ globin genes shared a **[7dpi↑-28dpi↑]** pattern. The failure to detect erythroid enriched *Gata*1 and *Eraf* mRNAs may reduce the likelihood of blood contamination as a source of *Hb* signal. Globin genes in non-erythroid tissue including neurons and glia are constitutively expressed or are induced following a cellular insult *eg* hypoxia [61]. There was an up-regulation of the neuronal specific *Ngb* **[7dpi↑]** and the ubiquitously expressed *Cygb* **[7dpi↑]** encoding neuroglobin and cytoglobin respectively that may provide neuroprotection against hypoxic attack [62], however, the physiological role of these neural globins remains equivocal [63]. Two participants of hypoxia *HIF signalling* ID^4066^ pathway, the endothelial specific transferrin receptor gene *Tfrc* necessary for iron uptake and the *Pdh*a1 gene encoding the key enzyme pyruvate dehydrogenase were down-regulated with attendant high FCs.

The activities of a small panel of myelin genes including the three high FC CNS myelin structural genes *Plp*1, *Mobp* and *Omg*, *Nsmaf*, a sphingomyelin enzymatic gene, a myelin transcription factor gene *Myt*1l and *Mal*1 involved in myelin sheath maintenance were decreased by 7dpi (S10 Table). The length of this transient dysregulation period may be sufficient to challenge the structural integrity of the CNS myelin sheath. Under physiological conditions, neurons are reliant on oligodendroglial support in ensuring the correct propagation of action potentials which may impact synaptic activity.

### Host immunity genes

Over 300 immune genes, the majority described either by the **[28dpi↑]** or the minor **[7dpi↑-28dpi↑]** expression profile were expressed during infection (S2 Table). Selected genes to represent the spectrum of the host’s response categorised into nine functional groupings are detailed in S11 Table. This revealed a panel of markers for both innate immunity (microglia (*Tmem*119 **[7dpi↑-28dpi↑]** and macrophages *Mpeg*1 **[28dpi↑]**) and adaptive immunity (the immunoproteasome gene *Psmb*8 **[7dpi↑-28dpi↑]** and the two high FC genes MHC class II *Cd*74 and the MHC I beta-2-microglobulin (β2M) (S11 Table). Genetic evidence of early diapedesis activity was inferred by the expression patterns of *Selpg* **[7dpi↑-28dpi↑]** encoding the E-selectin ligand and *Icam*2 **[7dpi↑-28dpi↑]** in addition to the Cam integrin genes *Itgb*4 and *Itga*7 **[7dpi↑]** (S11 Table). Of note was the activity and diverse function of three other CAM genes sharing the **[28dpi↑]** pattern. *Cd*274, with a high FC expresses the programmed death ligand-1 (PD-L1) leading to T cell suppression, *Cd*40 is a member of the *Tnfr* superfamily and *Cd*86 involved in T lymphocyte proliferation (S11 Table). The high activity level (26.4 FC) of the extrahepatic gene *Saa*3, encoding the serum amyloid acute phase protein SAA3 which is secreted into the CSF had a classic **[28dpi↑]** pattern may justify biomarker investigation as Stage2 biomarker as is the *Cd*74 gene (44.94 FC) (S11 Table).

Raised levels of IgM and IgG are recognised in *African trypanosomiasis* ID^5143^ (S1 Fig). Although 13 (14-28dpi)^9^ “immunoglobulin” GO: terms were up-regulated, no annotations specific to IgM or IgG activity were recorded. Indirect evidence of IgM activity was inferred from the expression kinetics of the *Mzb*1 gene **[28dpi↑]** which is involved with IgM assembly and secretion, but not IgG [64] while *Igj* **[28dpi↑]** encoding a polymerizing protein that links IgM monomers. There was an increase in the levels of the Fc gamma receptors genes (*Fcγr*), particularly *Fcgr*4 and *Fcgr*3 based on FC values and to a lesser extent *Fcgr*2b. These multifunctional *Fcγr* genes that share a **[28dpi↑]** pattern may be indicative of late IgG activity (S11 Table).

Expression of these 300 genes or so, underpinning both innate and adaptive immunity are in part orchestrated by a panel of documented cytokines and a network of chemokines as outlined below.

#### The cytokine gene expression profile

As noted previously, five GO terms were up-regulated by 7dpi (S4 Table) consistent with the common **[7dpi↑]** pattern for *Ifn* α/β, *Ifn*β1 and the cognate transcription factor gene *Irf*3 (S11 Table). An earlier study reported a role for Type I IFNs in early resistance [65] while the *Ifn* α/β receptor KO mouse had a reduced number of trypanosomes in the parenchyma compared to wild type [18]. In addition to *Ifn* α/β, the pro-inflammatory Th1 response comprised *Ifnγ*, *Tnf*α, *Il*1α, *Il*1β, and *Il*18, all with a **[28dpi↑]** profile. The inflammasome, a pathogen sensor mediates the activation of *caspases*-1 and 4 that cleave the pre-IL1 β and IL18 polypeptides to release the highly pro-inflammatory cytokines [66]. Both caspase genes, in addition to *Il*1β, and *Il*18 shared the incremental **[28dpi↑]** pattern. There was no evidence of a Th17 mediated pro-inflammatory response. Th2 anti-inflammatory activity was limited to *Tgfb*-1 **[7dpi↑-28dpi↑]** in addition to *Il*6 and *Il*10, both with a **[28dpi↑]** profile as had their corresponding receptor genes. Two other active interleukin genes were *Il*15 **[28dpi↑]** associated with GABA and serotonin transmission [67] and *Il*34 **[7dpi↑]** encoding a ligand for the microglia receptor CSF1R [68] had a **[7dpi↑-28dpi↑]** profile. Unlike the afore described cytokines with modest FCs, the *Ik* cytokine gene with a **[7dpi↓]** pattern had a high FC. The encoded nuclear spliceosomal RED protein represses the expression of (MHC) class II induced antigens by IFNγ [69]. A comparison of the cytokine gene expression profile described here with *African trypanosomiasis* ID^5143^ and a rodent brain compilation study [3] identified a string of common genes are highlighted in in shade (Table 4). Notable differences were the absence *Il*12a and *Il*12b from the rodent brain profiles and the absence of *Il*Ia from *African trypanosomiasis* ID^5143^.

**Table 4:** Comparison of cytokine gene expression profiles derived from this study, *African trypanosomiasis* ID^5143^ KEGG pathway and the Masocha compilation [3].

This study	***Ifnαβ***	***Ifnγ***	***Tnf*α**	***Il*1α**	***Il*1β**	***Il*18**		***Il*10**	***Tgfβ***			***Il*15**	***Il*34**	***Ik***
HAT KEGG		***Ifnγ***	***Tnf*α**		***Il*1β**	***Il*18**	***Il*6**	***Il*10**		***Il*12a**	***Il*12b**			
Masocha [3]	***Ifnαβ***	***Ifnγ***	***Tnf*α**	***Il*1α**	***Il*1β**		***Il*6**	***Il*10**	***Tgfβ***					

JAK-STAT activity initiates interferon cytokine ligand binding leading to the transcription of a myriad of key IFNα/β and IFNγ families of ISGs most with a **[28dpi↑]** or **[7dpi↑-28dpi↑]** pattern (S11 Table), several with a high FC. Prominent IFNα/β ISGs included members of the Interferon Regulatoryu Factor (*Irf*) family [70], the IFN-induced transmembrane protein (*Ifitms*) grouping [71] and the IFN-induced protein with tetratricopeptide repeats (*Ifit*) family [72]. The guanylate-binding protein GTPases families the small 47 kDa Immunity-Related GTPases and 65-67Kda family play a pivotal role in the type 2 interferon response. In addition, *Gvin* a member of a very high MW GTPase family of unknown function was active [73].

There was genetic evidence of Type 1 IFN suppression by the increased Stage 2 message levels of suppression marker genes *Cd*274, *Parp*14 *Trim*21 and *Usp*18 [74] with a common **[28dpi↑]** pattern. The activity of two genes *Socs*3 **[28dpi↑]** and *Pias*3h **[7dpi↓]** was altered which can lead to suppression of cytokine signalling by inhibition of the JAK-STAT pathway. The forkhead transcription factor gene *Foxp*3 **[7dpi↑]** has a critical role in Treg mediated suppression [75].

#### The chemokine gene expression profile

The main drivers of the chemokine gene response as measured by elevated FCs (S11 Table) were the *Ccl* members, *Ccl*4, *Ccl*5, *Ccl*7 and *Ccr*5 **[28dpi↑]**, the *Cxcl* species, *Cxcl*9, *Cxcl*10, *Cxcl*11, *Cxcr*3 *Cxcl*13 **[28dpi↑]**, *Cxcl*16 **[7dpi↑-28dpi↑]** and *Cxcl*14 **[7dpi↑].** The *Cxcl*10 post-14dpi inducible pattern and to lesser extent *Cxcl9* is consistent with the predicted high CXCL10 gradient in the astrocytic endfeet of the NVU purported to facilitate the coupled traversal of leukocytes and trypanosomes across the BBB [26]. Using the same end-point RT.PCR stratagem, we have reported a similar expression profile for the *Ifnγ* and inducible ligand genes *Cxcl*9, *Cxcl*10 and *Cxcl*11 and its shared receptor gene *Cxcr*3 to that reported in the infected brains of a rat model of HAT [34]. In contrast to the other *Cxcl* mRNAs, *Cxcl*14 had a Stage 1 **[7dpi↑]** signature while a recent study found that the pleiotropic CXCL14 specifically binds to CpG DNA and activates TLR9 on macrophages, thereby inducing inflammatory cytokines [76]. This expression signature was compared (Table 5) with a compilation profile of *Ccl* and *Cxcl* ligand genes expressed in infected rodent brains [3]. Common genes are depicted in shade, with the exception *Cxcl*11 which harbours a mutation in the C57/BL6 mouse other differences between the profiles was the presence of *Cxcl*1 and *Cxcl*5 in addition to the grouping of *Ccl* genes 9, 12, 19, 21 and 28 in the Masocha profile [3]. Although a four-fold increase in *Ccl*28 expression in the (6-28dpi) pairing was reported [30], no message activity was recorded. Other active chemokine genes not featured in the Masocha profile [3], were *Cx3cl*1 **[7dpi↑]** originally termed fractalkine in man, which unlike other chemokines has both chemoattractant and cell adhesion properties. Its cognate receptor gene *Cx3cr*1 had a **[28dpi↑]** pattern.

**Table 5:** Comparison of the chemokine ligand gene expression profiles derived from this study and Masocha compilation [3].

**Masocha [3]**	***Ccl* gene ligand family**	*Ccl*2	*Ccl*4	*Ccl*5	*Ccl*7	*Ccl*9	*Ccl*12	*Ccl*19	*Ccl*28	
**This study**	***Ccl* gene ligand family**	**(a)**	*Ccl*4	*Ccl*5	*Ccl*7	**(b)**	**(c)**	*Ccl*19	**(d)**	
**Masocha [3]**	***Cxcl* gene ligand family**	*Cxcl*1	*Cxcl*5	*Cxcl*9	*Cxcl*10	**(e)**	*Cxcl*12	*Cxcl*13	*Cxcl*14	*Cxcl*16
**This study**	***Cxcl* gene ligand family**	**(a)**	**(c)**	*Cxcl*9	*Cxcl*10	*Cxcl*11	*Cxcl*12	*Cxcl*13	*Cxcl*14	*Cxcl*16

**(a)**. [28dpi↑] Expression parameters sub-threshold

**(b)**. [7dpi↑-28dpi↑] Expression parameters sub-threshold

**(c)**. Probe not encoded on chip.

**(d)**. mRNA not detected.

**(e)** *Cxcl*11 gene is deleted in other mouse strains

As detailed earlier, although most of the cytokine and chemokine genes, in addition to many innate and adaptive immune genes had a **[28dpi↑]** or a **[7dpi↑-28dpi↑]** profiles, several transcripts increased incrementally between 0 and 14dpi evidence of a Stage1 immune response. This selected panel included the innate mRNAs, *Cxcl*9, *Cxcl*13 and *Ccl*5 and *H*2-*K*2, *H*2-*DMb*1and *B2m* as representative of adaptive immunity genes (S11 Table). This is consistent with the mixed neuronal-immune phenotype predicted from the functional enrichment analysis describing the period between 0 and 14dpi.

#### Brain barrier integrity/impairment genes

As laid out in S12 Table, candidate genes associated with barrier impairment can be broadly classified whether the genetic changes are **(a)** “disruptive”, involving alterations in the macromolecular architecture of the endothelia paracellular barrier complex or **(b)** non-disruptive or “functional” leading to an increase in vascular permeability caused by well documented and candidate mediators [77]. A useful resource reference for this screen was a “molecular atlas” [51] of brain enriched mRNAs assembled from a compilation study of control and disease BBB expression databases comprising 17 paracellular genes, eight members of the *Abc* transporter family and 39 genes belonging to the *Slc* transporter super family.

**(a)** *Disruptive changes*. Excluding the endothelial gene *Esam* **[7dpi↑]** encoding the endothelial specific adhesion JAM protein, this screen failed to detect significant changes in the activities of any of the key paracellular barrier structural genes consistent with the subthreshold values for the *Tight Junction* ID^4530^ and *Tight junction* GO:^0005923^. However, a set of ten ancillary including *Tight Junction* ID^4530^ genes *eg Ctnna*1 **[7dpi↑]**, *Tjap*1 **[7dpi↑]** and *Exoc*4 **[7dpi**↓**]** were altered by 7dpi (S12 Table). A late event during the neuroinvasion process is the enzymatic degradation of the basement membrane by the metalloproteinases, most notably MMP2 and MMP9 [25]. However, the significance of this to the corresponding expression patterns *Mmp*2 **[7dpi↑-28dpi↑]** and *Mmp*9 **[7dpi↑]** is equivocal as the expression parameters were subthreshold. The *African trypanosomiasis* ID^5143^ (S1 Fig) schematic highlights the importance of the laminin composition of the endothelial perivascular space during neuroinvasion. An immunofluorescence study observed that the ECM protein laminin 4 is permissive for parasite migration from the parenchymal basement while laminin 5 restricts entry into the parenchyma [24]. Despite having different expression profiles *Lama*4 **[7dpi↓]**and *Lama*5 **[7dpi↑]** (S12 Table), subthreshold expression parameters tempers comparisons between both studies.

**(b)** *Functional changes*. *African trypanosomiasis* ID^5143^ (S1 Fig) incorporates a non-disruptive functional neuroinvasion route based on an *in vitro* human endothelial layer BBB chamber model study [27]. It has been proposed that the trypanosome encoded cysteine protease brucipain binds to the membrane bound PAR2 receptor disrupting the host’s calcium signalling network facilitating the transendothelial migration underpinned by nine denoted genes, six of which were differentially expressed at 7dpi including the signal transduction gene *Gnaq* **[7dpi**↓**]** two phospholipase genes *eg Plbc*3 **[7dpi↑]** and three protein kinase C genes *eg Prkc*a **[7dpi**↓**]**. Further evidence of this critical relationship between maintenance of calcium homeostasis and BBB integrity [78] was the early down-regulation of around fifty calcium signalling genes. Of the 11 selected entries (S12 Table), *Cacnb*4, *Camk*, *Ednrb* and *Itpr*1 had high FCs in addition to *Slc*24a2 and *Slc*8a1 encoding calcium exchangers. This was reflected in a decrease in *Calcium signalling* ID^4020^ activity albeit at a marginal subthreshold value in addition to ten (0-7dpi)^1^ GO terms *eg Calcium ion transmembrane transport* GO:^0070588^ and the up-regulation of 18 (0-28dpi)^4^ terms possibly reflecting a different role for calcium post-14dpi.

Barrier homeostasis is regulated in part, by a cohort of pro- and anti-angiogenic mediators several of which were differentially expressed albeit with modest FCs by 7dpi (S12 Table). This included the up-regulation of three members of the VEGF/PDGF family [79], and the angiogenic genes *Angptl* 4 **[7dpi↑]**, *Angpt*l6 **[7dpi↑]**, the pro-angiogenic endothelial specific *Aggf*1 **[7dpi↓]** [80] and *Eng* [81] **[7dpi↑]**. In contrast to these mRNAs, an increase in the activities of, *Angpt*1 and the highly endothelial enriched *Angpt*2 increased post-14dpi [**28dpi↑]**. Dysfunctional signalling of the Wnt/beta catenin pathway can lead to BBB breakdown [82]. The four ligand genes *Wnt*2, *Wnt*4, *Wnt*7a and *Wnt*7b and its cognate receptor *Fzd*2 gene shared a **[7dpi↑]** pattern.

Perturbation in the expression of the *Abc* and *Slc* transporter gene families, particularly the latter, as alluded to elsewhere may challenge the functionality of the BBB. In addition, an increase in the low rate of endothelial transcytosis network during CNS disease could also impact on vascular permeability consistent with a modest up-regulation of three well characterized endothelial transcytosis genes, *viz Cav*1 [83] **[28dpi↑]**, *Mfs*d2 [84] **[7dpi↑]** and *Plvap* [85] **[7dpi↑]**. The activity of the highly endothelial enriched *Ly6a* message **[28dpi↑]** with an attendant high FC may be of interest. The gene encodes a GPI-anchored protein that facilitates the neuroinvasion of the adeno serotype AAV-PHP.B in a mouse strain specific manner, including CD1 [86]. The Aquaporin-4 water channel encoded by the *Aqp*4 gene **[7dpi↓]** was abundantly expressed in the astroglial endfeet has a central role in CNS water regulation linked to BBB permeability [87]. Both gene and the *Vasopressin-regulated water reabsorption* ID^94962^ pathway were down-regulated by 7dpi.

## Discussion

Disease progression over the 28 day timeline of the CD1/GVR35 HAT mouse has been well characterised in contrast to the underlying transcription profile. Based on this knowledge gulf, the twin aims of this microprofiling study were to **i)**: track changes to the quantitative and qualitative transcriptomes of infected CD1 mice brains leading to the identification of message populations and predicted phenotypic traits **ii)**: examine the expression profiles of candidate genes with documented or potential association with trypanosomiasis. Within the general context that the infected transcriptome could be broadly divided into four message population each with a distinct expression pattern, one critical and novel observation in addition to three others define this study. First, the period between 0 and 14dpi was marked by the coincident up-and down-regulation of over 8,000 transcripts approximating to around 4,000 genes. Functional enrichment analysis of this population identified many of the active genetic programmes to be associated with changes in neuronal behaviour and an early immune reaction. Second, the levels of over 300 mRNAs associated with various aspects of the immune response were induced post-14dpi. Third, although markers indicative of early changes in vascular permeability were identified by 7dpi, there was no genetic evidence of any structural disruption to the paracellular BBB. Fourth, candidate screening validated the expression profile of many documented immune response genes but failed to corroborate the activity of others associated neuropathogenesis and identified a small cohort of novel transcripts considered for further study.

It should be emphasised that before discussing the significance of these four findings, the interpretation of the microarray data *per se* should be tempered by two technical constraints. The first, is the inherent issue of the weighting to be given between differential expression p values and the corresponding fold change [36] as illustrated with the (0-7dpi)^1^ *Comparison*, where only 2370 transcripts had a FC± ≥2 from an active message population of 8145 mRNAs. The second, concerns the detection sensitivity when using whole brain homogenates where changes in message abundance in infected small regional loci *eg* BBB or SCN could be masked by the combined expression levels in the rest of the non-infected brain. This scenario may explain the failure to validate published data such as the endothelial basement laminin protein genes [24] and ECM metalloproteinase genes [25].

In contrast to that reported here, there was no evidence of an equivalent period of differential gene expression activity between 0 and 14dpi in C57/BL6 brains infected with the AnTat 1.1E *T*.*b brucei* stabilate [29]. Notwithstanding these inconsistencies during Stage 1 between the murine models, there was a similarity in gene numbers between the Amin study pairing (6-28dpi) [29] and our equivalent *Comparison* (7-28dpi)^4^ in addition to qualitative Stage2 similarities between the chemokine profiles [26] and in a cohort of non-cytokine/chemokine secreted proteins [30]. These transcriptomic differences may in part arise from the variation in the complex interplay between different combinations of host and parasite genetics. This is consistent with the variability in trypanotolerance between a series of commonly used inbred mouse model strains [6]. Gross differences were reported in spleen and liver pathology following infection of two *T*. *b*. *brucei* genetically distinct stabilates TREU927 and STIB247 in BALB/c mice [88]. Variation in trypanotolerance in the cattle breeds N’Dama and Boran infected with *T*. *congolense* [89] as well as differences in the disease profiles between *T*.*b brucei* and *T*.*b rhodesiense* patients, the development of CNS symptoms in *rhodesiense S*tage1 patients [14] and the presence of asymptomatic patients [90] all highlight the spectrum of responses elicited by this parasite. Moreover, an intermediate stage in HAT has been proposed following reported variation in the levels of the Stage 2 biomarkers CXCL13 and Neopterin in *T b gambiense* patients [91].

Functional enrichment analysis up to 14dpi identified genetic networks that predicted a myriad of transient changes to metabolism, mitochondrial activity, pleiotropic signalling, transporter activity, calcium signalling, neurotransmission including synaptic plasticity, circadian activity and vascular permeability. There was also evidence of a type I interferon response, activation of the innate system triggering both a B- and T-cell responses against trypanosome VSG coat antigens and complement activation. These phenotypic traits can be envisaged as the precursor for the initiation of a string of neuronal and immune changes that precede progression to Stage 2. These observations share similarities with the outcome of a *T*.*b*.*brucei* rat model collaborative study reporting the detection of trypanosome DNA at 6dpi, the presence of parasites and T cells in the parenchyma at 9dpi and significantly, early signs of circadian disruption [12]. Data from our CD1/GVR35 model [13] also detected trypanosome DNA in brain homogenates by 7dpi and observed significant barrier impairment on CE-MRI and neuroinflammation at 14dpi. Moreover, intravital microscopy observations claimed the presence of fluorescently tagged GVR35 trypanosomes within the parenchyma of infected mice as early as five hours after infection [11]. However, this whole issue of parenchymal invasion has been fundamentally challenged in a rat model that failed to detect trypanosomes in the parenchyma and contended that the parasites were restricted to the pial space following breakdown of the blood CSF barrier [15]. These combined studies purporting the early presence of parenchymal trypanosomes may be sufficient to elicit an early expansive genetic change consistent with our transcriptome and phenotypic observations up to 14dpi.

This period of early initiation of innate and adaptive immunity was followed by a secondary wave of post-14dpi activity marked by an up-regulation of over 300 immune genes including a combined grouping of immune-regulatory cytokine genes, ISG’s and chemokine genes (S11 Table) that control the innate response. The pro-and anti-inflammatory cytokine gene signatures are similar with a rodent model consensus profile [3]. There was no evidence of a pro-inflammatory Th17 response. A notable feature was an expansive increase in ISG activity comprising members the IFNα/β induced *Irf*, *Ifitm* and *Ifit* gene families and the IFNγ induced large 65- to 67-kDa GTPases and p47GTPase gene families. Message levels of ISGs *Irgm*3, *Ifi*47 and *Gbp*2 were also increased during *T*.*gondi* infections [73]. Excluding the *Cxcl*14 gene expression that peaked at 7dpi, the post-14dpi chemokine gene expression signature closely resembled a rodent model compilation profile [3]. This post-14dpi period was marked by the activity of many adaptive genes including histocompatibility H2 immunoproteasome genes supplemented by a large grouping of “Adaptive” KEGG and GO terms. Specificity to protozoan infection was confirmed by the up-regulation of six protozoan response KEGG pathways including the eponymous *African trypanosomiasis* ID^5143^. There was evidence of shift the immune response towards an increase in Type 1 IFN suppression inferred by the post 14dpi activity of the suppression marker genes *Cd*274, *Parp*14 *Trim*21 and *Usp*18 [74].

By CE-MRI, we were able to quantify an increase in contrast agent leakage signal at 14dpi [13]. However, it is unknown whether this change in permeability was “disruptive” or “functional” in origin [77]. There was no evidence of expression differences in any of the major paracellular complex genes or pathways. This is consistent with a rat study where trypanosomes were able to cross the BBB without any loss of tight junction proteins [22] although dysregulated tight junction activity was claimed to account for an increase in fluorescent dye uptake in a rat model [21]. Functional impairment could arise from the perturbed expression between 0 and 14dpi of any of the two BBB enriched *Abc* and nine *Slc* genes [51]. During this period there were fluctuations in genes that regulate calcium homeostasis [78] and angiogenesis [80–82] both processes being critical to barrier integrity. However, it remains equivocal whether any of these proposed early transient changes could impact on functional barrier activity to account for leakage of contrast agent from the BBB at 14dpi [13].

A recent RNASeq study [92] on transcriptomes from peripheral blood and the CSF of *T*.*b*. *rhodesiense* late Stage 2 patients reported a robust innate response in the blood transcriptome sharing genes and pathways catalogued in the (0-7dpi)^1^ and (7-14dpi)^7^ pairings. In contrast, the late CSF transcriptome had a different expression landscape reflected in the enrichment of anti-inflammatory and neuro-degeneration signalling pathways indicative of a more severe and advanced neurological phenotype than the 28dpi CD1 brain homogenate. Of additional interest were the differences between the *Cxcl* ligand gene profiles highlighted by the absence *Cxcl*9, 10, 11, 14 and 16 and the presence of *Cxcl*3, 5 and 13 in the CSF.

Candidate trypanosomiasis genes with high FCs include *Cd*74. Generally regarded a MHC class II chaperone, it has been shown to be multi-functional in pathological situations [93] and was expressed at high levels in the *Toxoplasma gondi* infected mouse brain [94]. Others include *Csrp*1 (neuronal development), *Cd*274 (T cell inhibition), *Ly*6a (neuroinvasion), three serpin genes *Serpina*3g, *Serpina*3h and *Serping*1 (multifunctional protease inhibitors), *Cxcl*14 (7dpi peak expression) and *Saa*3 as a Stage 2 biomarker. Others based on functionality are members of the multifunctional transporter *Slc* gene family involved in neurotransmission and BBB transport, several circadian genes *eg Gria*2, *Gria*3 and *Itpr*1 and the three BBB transcytosis genes *Cav*1, *Plvap* and *Mfsd*2 in addition to the *Ly*6a.

These changes to the neurotransmission network, the innate response, circadian activity and vascular permeability between 0 and 14dpi merit further investigation. Using the current experimental paradigm, with the inclusion of a series of additional time points either side of 7 and 14dpi, would provide more detailed temporal mapping of these key phenotypic changes and define the temporal lengths of the up- and down-regulated plateaus. A similar approach might be considered for more accurate immune mapping. This may also unravel late CNS disease genes and pathways identified in the late Stage 2 *rhodesiense* transcriptome study [92].

Future high throughput studies would be severely compromised using whole brain tissue. Further investigation requires a more ambitious experimental paradigm using advanced but established and readily accessible technologies such as Laser Capture Microdissection [95] or a single cell–omics approach [96]. This would facilitate access to distinct functional anatomical brain regions to generate homogenous transcriptomes and corresponding trypanosome DNA loads. This has recently been performed on different endothelia transcriptomes [97], in addition to the SCN [53] and the choroid plexus [98]. Adopting a RNASeq experiment paradigm to quantify homogenous cell populations coupled to the anticipated sensitivity advances in cell imaging and CE-MRI will extend our knowledge of when and by what route(s) the trypanosomes enter the CNS as well as reveal some of the molecular complexities of the phenotypic traits that describe neuroinvasion, the immune response and neurological dysfunction including disruption of the circadian network.

In conclusion, this study has reinforced the growing consensus that early neuronal and immuno phenotypic changes precede CNS disease. This justifies the case for further investigation of the up- down- regulation wave of activity up to 14dpi and has also provided a valuable reference resource, documenting the expression patterns of over 8000 altered transcripts.

## Supporting information

S1 Fig *African trypanosomiasis* ID^5143^ KEGG pathway. The schematic is a combined gene and phenotypic flow chart highlighting the IFNγ mediated initiation of the innate response involving the TLR9-MYD88 cascade and the adaptive B cell attack on the trypanosome VSG coat responses during Stage 1 followed by cytokine mediated breakdown of the BBB and the role for laminins in this process. An alternative neuroinvasion route is recognised involving disruption of calcium homoeostasis following endothelial binding of the trypanosome cysteine protease brucipain. Parasite invasion activates a series of Stage 2 neurological pathological changes proposing critical roles for apoptosis, NO, prostaglandins and tryptophan metabolism. These key events in HAT disease progression are depicted within the framework of the 28 day timeline of the CD1/GVR35 mouse model.
(TIF)

S2 Fig Heatmap of up-regulated KEGG pathways. (TIF)

S3 Fig Heatmap of down-regulated KEGG pathways. (TIF)

S4 Fig Heatmap up-regulated GO terms. (TIF)

S5 Fig Heatmap of down-regulated GO terms. **Common legend to heatmaps (S2-S5).** KEGG and GO functional enrichment analyses data are depicted in the four heatmaps (S2–S5 Figs). Up-regulated KEGG pathways and GO terms are in red while blue depicts the down-regulated data. The ten *Comparisons* are arranged along the X axis and KEGG pathways and GO terms lie on the Y axis. The number of displayed KEGG pathways and GO terms was restricted to 75, presented in decreasing order of p values. Phenotypic demarcation in both KEGG and GO profiles are boxed. Hierarchical clustering was applied to pathway and GO terms.
(TIF)

S1 Table List of normalized transcripts. Only probes detected at least once across the 45,281 probeset spotted on the Illumina MouseWG6_V2_0_R3_11278593_A Bead Chip. were retained for subsequent analysis. Following normalization to minimise variation gene expression measurement, 23,213 probes were designated as transcriptionally active. This collection of normalized transcripts is presented in alphabetical order alongside the corresponding log_2_ expression unit value for each probe at the four time points *viz* 7dpi, 14dpi, 21dpi and 28dpi for each of the three replicates denoted R1, R2 and R3 and three non-infected control samples equivalent to 0dpi.
(XLSX)

S2 Table Generation of transcriptome datasets. Ten pair wise *Comparisons* were performed on this active probeset. Expression difference between each sample pair was set at a strict statistically significant adjusted p value <0.001 with an attendant adaptive threshold fold change with associated significance statistics. The Infected Series compared the non-infected control equivalent to 0dpi against the four infected time points denoted (0-7dpi)^1^, (0-14dpi)^2^, (0-21dpi)^3^ and (0-28dpi)^4^. The Temporal Series measured activity changes between six different infected time point combinations *viz* (7-14dpi)^5^, (7-21dpi)^6^, (7-28dpi)^7^, (14-21dpi)^8^, (14-28dpi)^9^ and (21-28dpi)^10^. The resultant transcriptome database (S2 Table) set out for each pairing in order of decreasing p values and corresponding fold changes.
(XLSX)

S3 Table **KEGG functional enrichment analysis of (A) up-regulated and (B), down-regulated pathways.** Up-regulated (A) and down-regulated (B) KEGG functional enrichment analysis was performed on Infected Series *Comparisons* (0-7dpi)^1^, (0-21dpi)^3^ and (0-28dpi)^4^ and the Temporal Series *Comparisons* (7-14dpi)^5^, (7-21dpi)^6^, (7-28dpi)^7^ and (14-28dpi)^9^. Analysis of pairings (0-14dpi)^2^, (14-21dpi)^8^ and (21-28dpi)^10^ were excluded as there was no significant gene expression activity in theses *Comparisons*. KEGG pathways/ID number are set out or each *Comparison* in order of decreasing order.
(XLSX)

S4 Table **GO Functional enrichment analysis of (A) up-regulated GO terms and (B), down-regulated GO terms.** Up-regulated (A) and down-regulated (B) KEGG functional enrichment analysis was performed on Infected Series *Comparisons* (0-7dpi)^1^, (0-21dpi)^3^ and (0-28dpi)^4^ and the Temporal Series *Comparisons* (7-14dpi)^5^, (7-21dpi)^6^, (7-28dpi)^7^ and (14-28dpi)^9^. Analysis of pairings (0-14dpi)^2^, (14-21dpi)^8^ and (21-28dpi)^10^ were excluded as there was no significant gene expression activity in theses *Comparisons*. KEGG pathways/ID number are set out or each *Comparison* in order of decreasing order.
(XLSX)

S5 Table Expression profiles of the top 20 differentially expressed genes in selected *Comparisons* (0-7dpi)^1^, (7-14dpi)^5^, (0-28dpi)^4^ and (14-28dpi)^9^. (DOCX)

S6 Table KEGG functional enrichment analysis of up-and down-regulated genes in (0-7dpi)^1^ charting phenotypic change by grouping pathways into broad functional categories. (DOCX)

S7 Table KEGG functional enrichment analysis of up-regulated genes in Temporal *Comparisons* (7-14dpi)^5^, (7-21dpi)^6^ and (7-28dpi)^7^ charting phenotypic change by grouping pathways into broad functional categories. (DOCX)

S8 Table KEGG functional enrichment analysis of up-regulated genes of *Comparisons* (0-21dpi)^3^ and (0-28dpi)^4^ identifying immune phenotypic traits by grouping pathways into a range of immune functional categories. (DOCX)

S9 Table **a) *African trypanosomiasis* ID**^**5143**^ **and b) Stage 2 Biomarker gene expression profiles**. The expression profiles of the 38 genes that define the *African trypanosomiasis* ID^5143^ KEGG pathway (a) and a panel of published HAT biomarker genes (b). Each gene was matched against its *Comparison* ^#^ with the maximum fold change (Max FC^#^), adj p value and expression pattern (Fig 3.).
(DOCX)

S10 Table Candidate genes associated with pathogenesis. Pathogenic candidate genes, were selected based on a high FC, inferred biological significance or both, were grouped into 13 functional categories. Each gene was matched against its *Comparison* ^#^ with the maximum fold change (Max FC^#^), adj p value and expression pattern (Fig 3.).
(DOCX)

S11 Table Host’s immune gene response. Immune genes were selected based on a high FC, inferred biological significance or both, were grouped into nine immune functional categories. Each gene was matched against its *Comparison* ^#^ with the maximum fold change (Max FC^#^), adj p value and expression pattern (Fig 3.).
(DOCX)

S12 Table Candidate genes associated with BBB integrity/impairment. Candidate genes were designated **a)** as “disruptive” involving structural changes to the paracellular barrier complex or b) “functional” where a change in the activity of a range of non-cytokine mediators. Each gene was matched against its *Comparison* ^#^ with the maximum fold change (Max FC^#^), adj p value and expression pattern (Fig 3.).
(DOCX)
