# Supplementary figures and images for: Microarray profiling predicts early neurological and immune phenotypic traits in advance of CNS disease during disease progression in Trypanosoma. b. brucei infected CD1 mouse brains
Source: PLoS Negl Trop Dis. 2021 Nov 11;15(11):e0009892. doi: 10.1371/journal.pntd.0009892 (PMC8584711; doi:10.1371/journal.pntd.0009892)

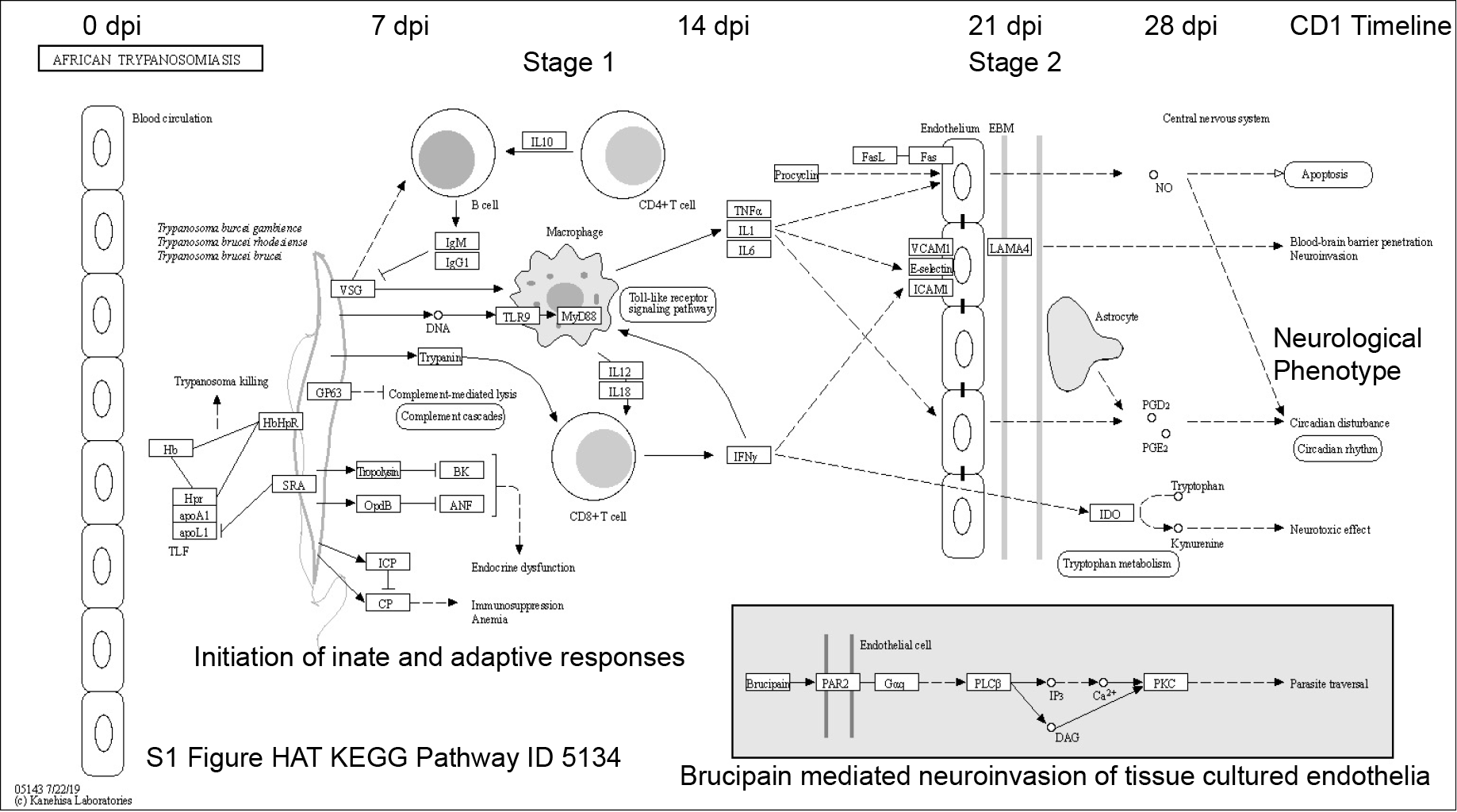

Supplement: S1 Fig — The schematic is a combined gene and phenotypic flow chart highlighting the IFNγ mediated initiation of the innate response involving the TLR9-MYD88 cascade and the adaptive B cell attack on the trypanosome VSG coat responses during Stage 1 followed by cytokine mediated breakdown of the BBB and the role for laminins in this process. An alternative neuroinvasion route is recognised involving disruption of calcium homoeostasis following endothelial binding of the trypanosome cysteine protease brucipain. Parasite invasion activates a series of Stage 2 neurological pathological changes proposing critical roles for apoptosis, NO, prostaglandins and tryptophan metabolism. These key events in HAT disease progression are depicted within the framework of the 28 day timeline of the CD1/GVR35 mouse model. (TIF) [file pntd.0009892.s001.tif]

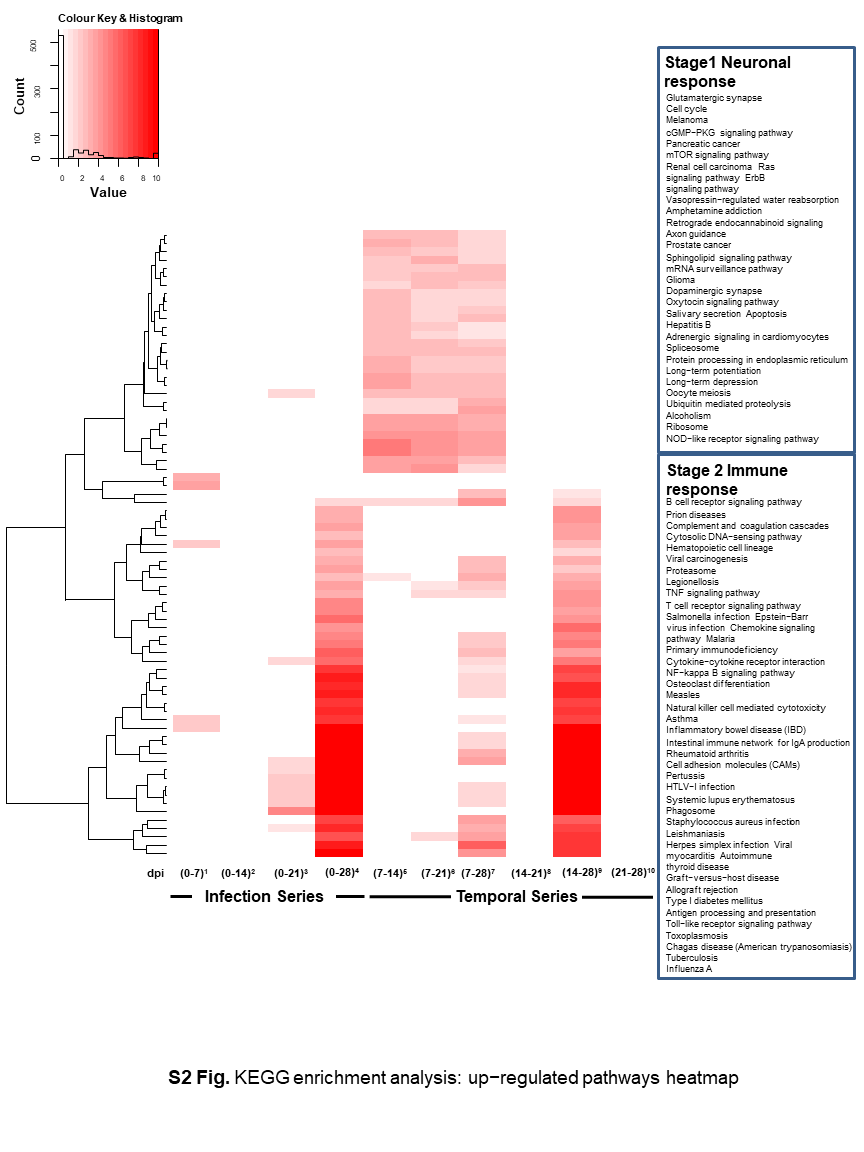

Supplement: S2 Fig — (TIF) [file pntd.0009892.s002.tif]

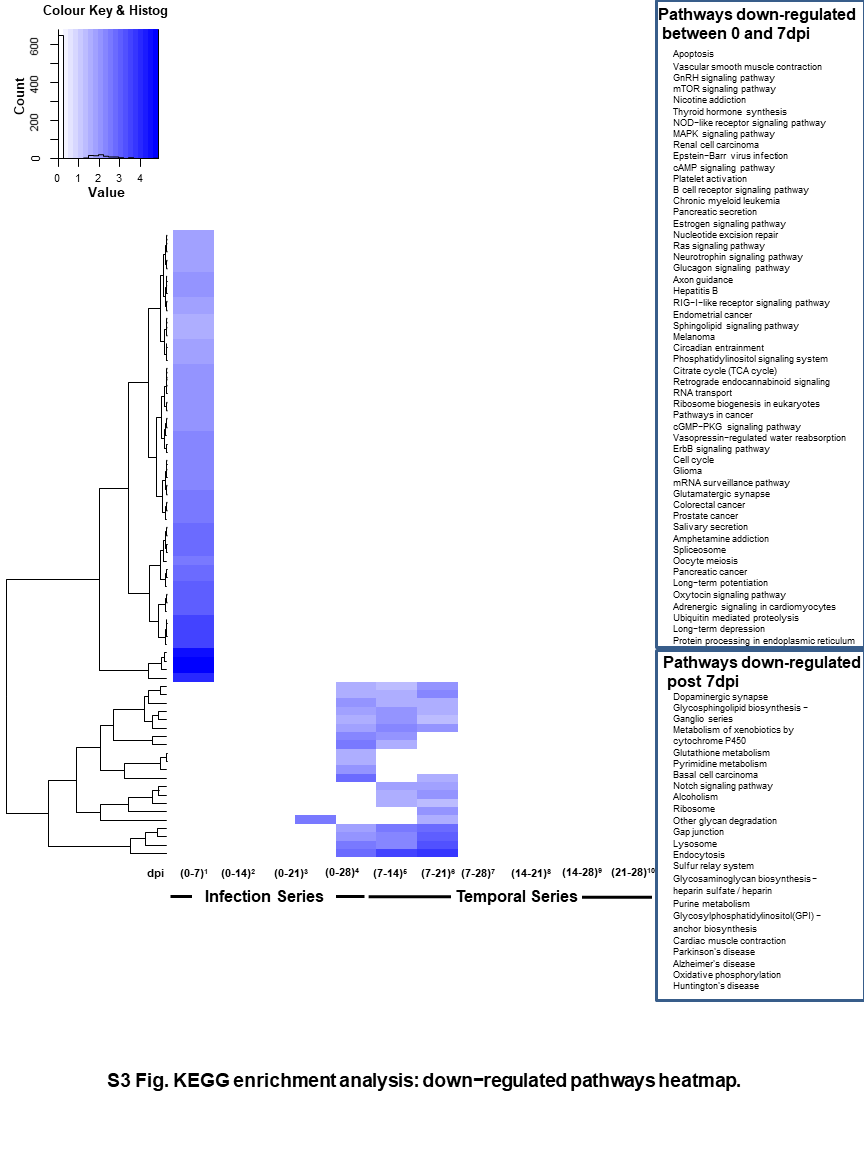

Supplement: S3 Fig — (TIF) [file pntd.0009892.s003.tif]

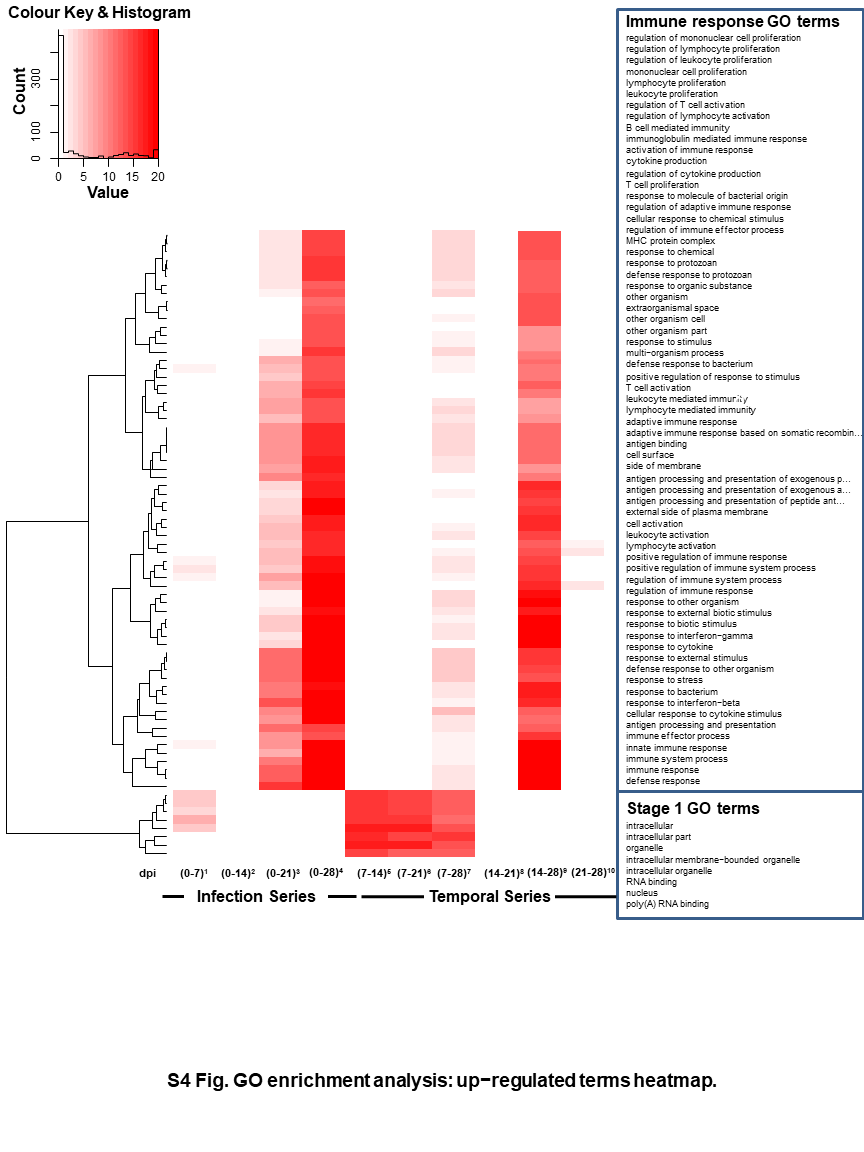

Supplement: S4 Fig — (TIF) [file pntd.0009892.s004.tif]

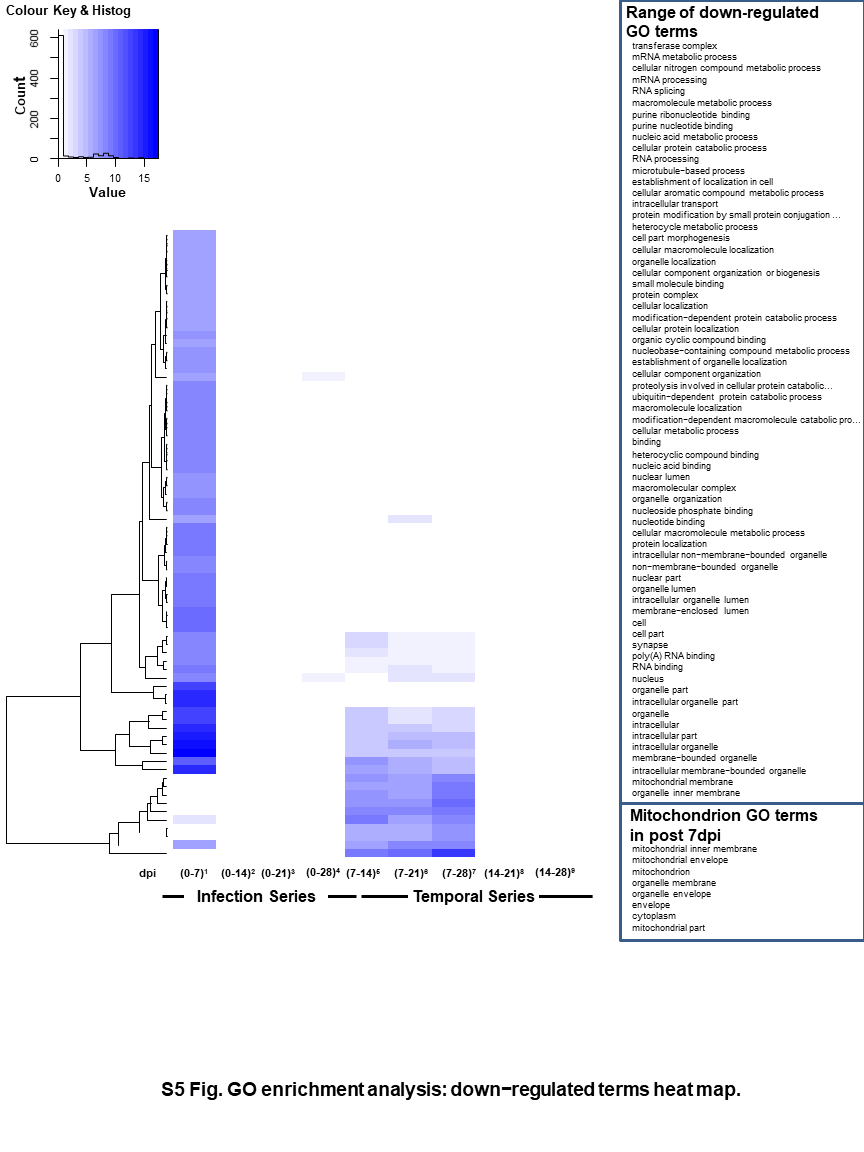

Supplement: S5 Fig — Common legend to heatmaps (S2-S5). KEGG and GO functional enrichment analyses data are depicted in the four heatmaps (S2–S5 Figs). Up-regulated KEGG pathways and GO terms are in red while blue depicts the down-regulated data. The ten Comparisons are arranged along the X axis and KEGG pathways and GO terms lie on the Y axis. The number of displayed KEGG pathways and GO terms was restricted to 75, presented in decreasing order of p values. Phenotypic demarcation in both KEGG and GO profiles are boxed. Hierarchical clustering was applied to pathway and GO terms. (TIF) [file pntd.0009892.s005.tif]
