# Supplementary material for: Microarray profiling predicts early neurological and immune phenotypic traits in advance of CNS disease during disease progression in Trypanosoma. b. brucei infected CD1 mouse brains
Source: PLoS Negl Trop Dis. 2021 Nov 11;15(11):e0009892. doi: 10.1371/journal.pntd.0009892 (PMC8584711; doi:10.1371/journal.pntd.0009892)
Supplement: S5 Table — (DOCX) [file pntd.0009892.s010.docx]

**S5 Table. Non-statistically filtered ±FC range for each *Comparison*.**

**a)**

| Comparison^#^ | Transcript N^os^ ↑ | 2x↑ | 4x↑ | 8x↑ | 16x↑ | 32x↑ | Transcript N^os^↓ | 2x↓ | 4x↓ | 8.0x↓ | 16x↓ | ↓32x↓ |
| --- | --- | --- | --- | --- | --- | --- | --- | --- | --- | --- | --- | --- |
|  |  |  |  |  |  |  |  |  |  |  |  |  |
| (0-7dpi)^1^ | 4360 | 740 | 23 | 2 | 1 | 1 | 3785 | 1713 | 295 | 21 | 1 | 0 |
| (0-14dpi)^2^ | 0 | 48 | 9 | 1 | 0 | 0 | 0 | 4 | 0 | 0 | 0 | 0 |
| (0-21dpi)^3^ | 37 | 121 | 39 | 8 | 2 | 0 | 5 | 9 | 0 |  | 0 | 0 |
| (0-28dpi)^4^ | 324 | 269 | 100 | 46 | 18 | 8 | 30 | 12 | 1 |  | 0 | 0 |
| (7-14dpi)^5^ | 3892 | 1760 | 322 | 23 | 0 | 0 | 4628 | 856 | 2 | 1 | 1 | 0 |
| (7-21dpi)^6^ | 4042 | 1885 | 340 | 31 | 1 | 0 | 4855 | 1061 | 3 | 1 | 0 | 0 |
| (7-28dpi)^7^ | 4008 | 1844 | 370 | 40 | 9 | 3 | 4951 | 1158 | 6 | 3 | 0 | 0 |
| (14-21dpi)^8^ | 0 | 63 | 5 | 0 | 0 | 0 | 0 | 10 | 0 | 0 | 0 | 0 |
| (14-28dpi)^9^ | 208 | 225 | 66 | 15 | 8 | 4 | 0 | 11 | 2 | 2 | 2 | 0 |
| (21-28dpi)^10^ | 39 | 83 | 24 | 8 | 4 | 0 | 0 | 10 | 2 | 1 | 0 | 0 |
