# Supplementary material for: Microarray profiling predicts early neurological and immune phenotypic traits in advance of CNS disease during disease progression in Trypanosoma. b. brucei infected CD1 mouse brains
Source: PLoS Negl Trop Dis. 2021 Nov 11;15(11):e0009892. doi: 10.1371/journal.pntd.0009892 (PMC8584711; doi:10.1371/journal.pntd.0009892)
Supplement: S6 Table — (DOCX) [file pntd.0009892.s011.docx]

**S6 Table.** **KEGG functional enrichment analysis of up-and down-regulated genes in (0-7dpi)^1^ charting phenotypic change by grouping pathways into broad functional categories.**

| Up-regulated (0-7dpi)^1^ |  | Down-regulated (0-7dpi)^1^ |  |
| --- | --- | --- | --- |
| (12/12) KEGG Pathway ID^No^ |  | **(40/67) KEGG Pathway ID^No^** |  |
|  |  |  |  |
| Mitochondrial Activity | **p-value** | **Transcriptionj** | **p-value** |
| Ribosome ID^3010^ | 1.85E-04 | Spliceosome ID^3040^ | 7.47E-04 |
| Oxidative Phosphorylation ID^0190^ | 5.64E-02 | Ribosome biogenesis ID^3008^ | 3.51E-03 |
| Neuronal dysfunction |  | RNA transport ID^3013^ | 3.65E-03 |
| Huntington's disease ID^5016^ | 2.49E-02 | mRNA surveillance ID^3015^ | 1.51E-03 |
| Alcoholism ID^5034^ | 4.23E-04 | **Protein processing** |  |
| Glycosphingolipid biosynth ID^604^ | 5.43E-03 | Protein processing in ER ID^4141^ | 1.25E-05 |
| Immune response |  | Ubiquitin mediated proteolysis ID^4120^ | 2.25E-05 |
| SLS (Lupus) ID^5322^ | 4.25E-03 | **Neurotransmission** |  |
| HTLV-I infection ID^5166^ | 7.53E-03 | Dopaminergic synapse ID^4728^ | 1.00E-04 |
| Metabolism |  | Glutamatergic synapse ID^4724^ | 2.14E-03 |
| Pyrimidine metabolism ID^240^ | 1.31E-02 | Serotonergic synapse ID^4726^ | 4.31E-02 |
| Glutathione metabolism ID^480^ | 1.38E-02 | SNARE in vesicular transport ^ID4130^ | 2.41E-02 |
| Metabolism of xenobiotics ID^980^ | 3.46E-02 | Synaptic vesicle cycle ID^4721k^ | 5.57E-02 |
| Pleiotropic signalling |  | Amphetamine addiction ID^5031^ | 7.80E-04 |
| Prolactin signalling ID^4917^ | 4.57E-02 | Endocannabinoid signalling ID^7423^ | 3.99E-03 |
| Notch signalling ID^4330^ | 2.47E-02 | Nicotine addiction ID^5033^ | 1.49E-02 |
| Endocytosis ID^4144^ | 1.27E-02 | **Synaptic plasticity** |  |
|  |  | Long-term depression ID^4730^ | 1.68E-05 |
|  |  | Long-term depression ID^4730^ | 1.68E-05 |
|  |  | **Neuronal activity** |  |
|  |  | Salivary secretion ID^4970^ | 1.09E-03 |
|  |  | Axon guidance ID^4360^ | 9.24E-03 |
|  |  | **Circadian activity** |  |
|  |  | Circadian entrainment ID^4713^ | 3.21E-04 |
|  |  | **Pleiotropic signalling** |  |
|  |  | Neurotrophin signalling ID^4722^ | 7.98E-03 |
|  |  | Calcium signalling ID^4020^ | 7.18E-02 |
|  |  | MAPK signalling ID^4010^ | 1.09E-02 |
|  |  | cAMP Signalling ID^4024^ | 2.05E-02 |
|  |  | ErbB signalling ID^4012^ | 1.84E-03 |
|  |  | Phosphatidylinositol signalling ID^4070^ | 4.33E-03 |
|  |  | cGMP-PKG signalling ID^4022^ | 3.15E-03 |
|  |  | Sphingolipid signalling ID^4071^ | 5.89E-03 |
|  |  | mTor signalling ID^4150^ | 1.56E-02 |
|  |  | Ras signalling ID^4014^ | 7.71E-03 |
|  |  | Rap1 signalling ID^4015^ | 3.63E-02 |
|  |  | **Apoptosis** |  |
|  |  | ApoptosisID^4210^ | 1.39E-02 |
|  |  | **Secretory endocrine** |  |
|  |  | Vasopressin water reabsorption ID^4692^ | 2.79E-03 |
|  |  | Glucagon signalling ID^4922^ | 7.43E-03 |
|  |  | Oxytocin signalling ID^4921^ | 9.24E-03 |
|  |  | Gnrh signalling ID^4912^ | 1.37E-02 |
|  |  | **Immune activity** |  |
|  |  | Rig1-like receptor signalling ID^4622^ | 6.64E-03 |
|  |  | Nod like receptor signalling ID^4621^ | 1.02E-02 |
|  |  | B cell receptor signalling ID^4662^ | 2.11E-02 |
|  |  | T cell receptor signalling ID^4660^ | 5.82E-02 |
|  |  | **Cell cycle** |  |
|  |  | Cell cycle ID^4110^ | 1.74E-03 |
