# Supplementary material for: Microarray profiling predicts early neurological and immune phenotypic traits in advance of CNS disease during disease progression in Trypanosoma. b. brucei infected CD1 mouse brains
Source: PLoS Negl Trop Dis. 2021 Nov 11;15(11):e0009892. doi: 10.1371/journal.pntd.0009892 (PMC8584711; doi:10.1371/journal.pntd.0009892)
Supplement: S7 Table — (DOCX) [file pntd.0009892.s012.docx]

**S7 Table.** **KEGG functional enrichment analysis of up-regulated genes in Temporal *Comparisons* (7-14dpi)^5^, (7-21dpi)^6^ and (7-28dpi)^7^ charting phenotypic change by grouping pathways into broad functional categories.**

| Comparison (7-14dpi)^5^ (25/70) | | Comparison (7-21dpi)^6^ (25/54) | | Comparison (7-28dpi)^7^ (25/80) | |
| --- | --- | --- | --- | --- | --- |
| KEGG Pathway ID^#^ | **p-value** | **KEGG Pathway ID^#^** | **p-value** | **KEGG Pathway ID^#^** | **p-value** |
|  | | | | | |
| Neurotransmission |  | **Neurotransmission** |  | **Neurotransmission** |  |
| Dopaminergic synapse ID^4728^ | 2.55E-04 | Glutamatergic synapse ID^4724^ | 1.28E-03 | Dopaminergic synapse ID^4728^ | 1.11E-03 |
| Glutamatergic synapse ID^4724^ | 1.07E-03 | Dopaminergic synapse ID^4728^ | 1.64E-03 | **Synaptic plasticity** |  |
| Serotonergic synapse ID^4726^ | 2.56E-02 | Amphetamine addiction ID^5031^ | 1.03E-02 | Long-term potentiation ID^4720^ | 1.15E-04 |
| GABAergic ID^4917^ | 6.55E-02 | Axon guidance ID^4360^ | 1.18E-02 | Long-term depression ID^4730^ | 2.02E-04 |
| Axon guidance ID^4360^ | 1.25E-03 | **Synaptic plasticity** |  | **Circadian activity** |  |
| Synaptic plasticity |  | Long-term potentiation ID^4270^ | 8.61E-05 | Circadian entrainment ID^4713^ | 4.49E-02 |
| Long-term depression ID^4730^ | 4.83E-06 | Long-term depression ID^4730^ | 4.75E-05 | **Apoptosis** |  |
| Long-term potentiation ID^4720^ | 9.48E-06 | **Circadian activity** |  | Apoptosis ID^4210^ | 5.93E-04 |
| Circadian activity |  | Circadian entrainment ID^4713^ | 2.08E-02 | **Pleiotropic signalling** |  |
| Circadian entrainment ID^4713^ | 5.22E-03 | **Cellular regulation** |  | HIF-1 signalling ID^4066^ | 4.13E-03 |
| Cellular regulation |  | ER Protein processing D^4142^ | 6.56E-05 | **BBB activity** |  |
| Protein processing in ER ID^4141^ | 7.29E-05 | Ubiquitin proteolysis ID^4120^ | 9.93E-05 | VEGF signalling ID^4370^ | 1.18E-02 |
| RNA transport ID^3013^ | 1.27E-02 | RNA transport ID^3013^ | 4.55E-02 | Wnt signalling ID^4310^ | 6.50E-02- |
| Apoptosis |  | Spliceosome ID^3040^ | 2.54E-04 | **Immune response** |  |
| Apoptosis ID^4210^ | 1.46E-02 | **Apoptosis** |  | B cell signalling ID^4662^ | 3.28E-05 |
| Pleiotropic signalling |  | Apoptosis ID^4210^ | 1.32E-02 | T cell signalling ID^4660^ | 7.12E-04 |
| ErbB signalling ID^4012^ | 1.95E-03 | **Pleiotropic signalling** |  | TLR signalling ID^4620^ | 2.76E-04 |
| Sphingolipid signalling ID^4071^ | 1.53E-03 | ErbB signalling ID^4012^ | 1.88E-04 | NF-k B signalling ID^4064^ | 8.04E-04 |
| Ras signalling ID^4014^ | 2.90E-03 | Adrenergic signalling ID^4261^ | 2.54E-04 | Tuberculosis ID^5152^ | 6.48E-07 |
| mTOR signalling ID^4150^ | 6.97e-03 | cGMP-PKG signalling ID^4962^ | 6.91E-04 | Influenza A ID^5164^ | 3.24E-05 |
| Neurotrophin signallingID^4722^ | 1.57E-02 | HIF SignallingID^4066^ | 6.54E-03 | Hepatitis B ID^5161^ | 3.11E-04 |
| MAPK SignallingID^4010^ | 1.94E-02 | Sphingolipid signalling ID^4071^ | 1.92E-03 | Legionellosis ID^5134^ | 1.21E-03 |
| GnRH signalling ID^4912^ | 2.76E-02 | mTOR signalling ID^4150^ | 2.05E-03 | Measles ID^5162^ | 1.77E-03 |
| HIF SignallingID^4066^ | 3.99E-02 | Neurotrophin signallingID^4722^ | 3.18E-02 | Salmonella infection ID^5132^ | 3.48E-03 |
| FoxO signalling ID^4068^ | 4.88E-02 | **Immune response** |  | Staph aureus ID^5150^ | 2.79E-02 |
| Immune response |  | B cell signalling ID^4662^ | 1.88E-02 | HTLV-1 infection ID^5166^ | 3.73E-02 |
| B cell signalling ID^4662^ | 2.21E-02 | Chagas disease ID^5142^ | 3.09E-02 | Graft-Vs-host ID^5332^ | 2.15E-02 |
| T cell receptor signalling ID^4660^ | 3.53E-02 | Epstein-Barr infection ID^5169^ | 3.10E-02 | Toxoplasmosis ID^5145^ | 3.73E-04 |
| BBB activity |  | Salmonella infection ID^5132^ | 4.89E-02 | Leishmaniasis ID^5140^ | 4.55E-04 |
| VEGF signalling ID^4370^ | 2.92E-02 | **BBB activity** |  | Chagas disease ID^5142^ | 1.04E-04 |
| Vasopressin reabsorption ID^4962^ | 2.93E-02 | Vasopressin reabsorption ID^4962^ | 1.35E-02 | African trypanosomiasis ID^5143^ | 4.75E-02 |
| Wnt signalling ID^4310^ | 7.28E-02 | VEGF signalling ID^4370^ | 8.79E-02 |  |  |
