# Supplementary material for: Microarray profiling predicts early neurological and immune phenotypic traits in advance of CNS disease during disease progression in Trypanosoma. b. brucei infected CD1 mouse brains
Source: PLoS Negl Trop Dis. 2021 Nov 11;15(11):e0009892. doi: 10.1371/journal.pntd.0009892 (PMC8584711; doi:10.1371/journal.pntd.0009892)
Supplement: S8 Table — (DOCX) [file pntd.0009892.s013.docx]

**S8 Table.** **KEGG functional enrichment analysis of up-regulated genes of *Comparisons* (0-21dpi)^3^ and (0-28dpi)^4^ identifying immune phenotypic traits by grouping pathways into a range of immune functional categories.**

| Comparison (0-21dpi)^3^(12/12) |  | Comparison (0-28dpi)^4^ (25/50) |  |
| --- | --- | --- | --- |
| KEGG Pathway ID^#^ | **p-value** | **KEGG Pathway ID^#^** | **p-value** |
|  |  |  |  |
| Innate activity |  | **Innate activity** |  |
| NK cell cytotoxicity ID^4650^ | 2.40E-02 | Complement & coagulation ID^4610^ | 4.57E-04 |
| Adaptive activity |  | Jak-STAT signalling ID^4630^ | 8.40E-03 |
| Antigen processing/presentation ID^4612^ | 2.65E-05 | NF-k- B signalling ID^4064^ | 1.15E-05 |
| CAMs ID^4514^ | 5.05E-02 | Cytosolic DNA sensing ID^6423^ | 1.71E-04 |
| Phagosome ID^4145^ | 5.53E-02 | TLR signalling ID^4620^ | 6.35E-08 |
| Infection |  | **Adaptive activity** |  |
| HSV infection ID^5168^ | 1.13E-02 | Antigen processing/presentation ID^4612^ | 6.73E-16 |
| Viral myocarditis ID^5416^ | 1.08E-02 | Hemopoietic cell lineage ID^4640^ | 1.56E-03 |
| Protozoan infection |  | T cell signalling ID^4660^ | 1.11E-03 |
| Toxoplasmosis ID^5154^ | 3.76E-02 | B cell signalling ID^4662^ | 1.60E-02 |
| Dysfunctional immunity |  | **Immuno-signalling** |  |
| Allograft rejection ID^5330^ | 4.52E-03 | TNF signalling ID^4668^ | 2.92E-04 |
| Graft-Vs-host ID^5332^ | 4.81E-03 | Chemokine signalling ID^4062^ | 3.03E-05 |
| Autoimmune thyroid ID^4940^ | 5.70E-03 | Cytokine-cytokine ID^4060^ | 3.54E-05 |
| Type I diabetes ID^4940^ | 7.35E-03 | **Viral/Bacterial Infection** |  |
| Sympathetic activity |  | Herpes Simplex infection ID^5168^ | 1.04E-14 |
| Salivary secretion ID^4970^ | 1.44E-02 | Staph aureus ID^5150^ | 4.24E-16 |
|  |  | Salmonella infection ID^5132^ | 1.97E-04 |
|  |  | Legionellosis ID^5134^ | 6.75E-04 |
|  |  | **Protozoan infection** |  |
|  |  | Leishmaniasis ID^5140^ | 1.35-14 |
|  |  | Toxoplasmosis ID^5145^ | 9.62E-09 |
|  |  | Chagas disease ID^5142^ | 1.76E-07 |
|  |  | Malaria ID^5144^ | 1.51E-05 |
|  |  | African trypanosomiasis ID^5143^ | 3.06E-03 |
|  |  | Amoebiasis ID^5146^ | 1.56E-02 |
|  |  | **Dysfunctional immunity** |  |
|  |  | SLE (Lupus) ID^5322^ | 1.32E-15 |
|  |  | **Diapedesis** |  |
|  |  | CAMs ID^4514^ | 1.26E-08 |
|  |  | Leukocyte transendothelial migration ID^4670^ | 3.13E-02 |
