# Supplementary material for: Microarray profiling predicts early neurological and immune phenotypic traits in advance of CNS disease during disease progression in Trypanosoma. b. brucei infected CD1 mouse brains
Source: PLoS Negl Trop Dis. 2021 Nov 11;15(11):e0009892. doi: 10.1371/journal.pntd.0009892 (PMC8584711; doi:10.1371/journal.pntd.0009892)
Supplement: S9 Table — a) African trypanosomiasis ID5143 and b) Stage 2 Biomarker gene expression profiles. The expression profiles of the 38 genes that define the African trypanosomiasis ID5143 KEGG pathway (a) and a panel of published HAT biomarker genes (b). Each gene was matched against its Comparison # with the maximum fold change (Max FC#), adj p value and expression pattern (Fig 3.). (DOCX) [file pntd.0009892.s014.docx]

**S9 Table** **a) *African trypanosomiasis* ID^5143^ and b) Stage 2 Biomarker gene expression profiles**.

a)

| *African trypanosomiasis* ID^5143^ Genes | Symbol | Max FC^#^ | Adj p-value | Pattern | Gene function |
| --- | --- | --- | --- | --- | --- |
| Myeloid differentiation response gene 88 | *Myd*88 | 1.74^4^ | 3.61E-02 | [7dpi↑-28dpi↑] | Innate cascade |
| Toll receptor 9 | *Tlr*9 | PCR^+ve^ |  | [28dpi↑] | Innate cascade |
| Thimet oligopeptidase 1 | *Thop*1 | -2.52^7^ | 1.21E-07 | [7dpi↑] | Circadian activity |
| Indoleamine 2,3-dioxygenase 1 | *Ido*1 | 1.40^7^ | 3.08E-05 | [28dpi↑] | Tryptophan metabolism |
| Indoleamine 2,3-dioxygenase 2 | *Ido*2 |  | Not detected |  | Tryptophan metabolism |
| Kininogen 1 | *Kng*1 |  | Not detected |  | Endocrine dysfunction |
| Kininogen 2 | *Kng*2 |  | Not detected |  | Endocrine dysfunction |
| Natriuretic peptide type A | *Nppa* | -1.40^7^ | 3.27E-05 | [7dpi↑] | Endocrine dysfunction |
| Apolipoprotein A1 | *Apoa*1 |  | Subthreshold | Invariant | Trypanolytic activity |
| Haemoglobin alpha, adult chain 2 | *Hba*-a2 |  | Not detected |  | Haemoglobin synthesis |
| Haemoglobin alpha, adult chain 1 | *Hba*-a1 | 3.34^9^ | 2.23E-02 | [7dpi↑-28dpi↑] | Haemoglobin synthesis |
| Haemoglobin beta adult major chain | *Hbb*-b1 |  | Not detected |  | Haemoglobin synthesis |
| Haemoglobin beta adult minor chain | *Hbb*-b2 | 1.42^9^ | 2.48E-01 | [7dpi↑-28dpi↑] | Haemoglobin synthesis |
| Haemoglobin beta adult t chain | *Hbb*-bt | 3.44^9^ | 4.06E-02 | [7dpi↑-28dpi↑] | Haemoglobin synthesis |
| Haemoglobin beta adult s chain | *Hbb*-bs | 3.38^9^ | 3.86E-02 | [7dpi↑-28dpi↑] | Haemoglobin synthesis |
| Interleukin 10 | *Il*10 | PCR^+ve^ |  | [28dpi↑] | Th2-cytokine |
| Interleukin 12a | *Il*12a |  | Subthreshold | Invariant | Th1 cytokine |
| Interleukin 12b | *Il*12b |  | Not detected |  | Th1 cytokine |
| Interferon gamma | *Ifn*g | 1.26^4^ | 6.24E-05 | [28dpi↑] | Th1 cytokine |
| Tumour necrosis factor | *Tnf* | PCR^+ve^ |  | [28dpi↑] | Th1 cytokine |
| Interleukin 1 beta | *Il1*β | 2.27^4^ | 2.53E-04 | [28dpi↑] | Th1 cytokine |
| Interleukin 6 | *Il*6 | PCR^+ve^ |  | [28dpi↑] | Th2-cytokine |
| Interleukin 10 | *Il*10 | PCR^+ve^ |  | [28dpi↑] | Th2-cytokine |
| Fas ligand (TNF superfamily, member 6) | *Fasl* |  | Subthreshold | [7dpi↑-28dpi↑] | B cell apoptosis |
| Fas (TNF receptor superfamily member 6) | *Fas* | 1.84^7^ | 1.01E-05 | [0-28dpi↑] | B cell apoptosis |
| Vascular cell adhesion molecule 1 | *Vcam*1 | 4.04^5^ | 1.07E-06 | [0-28dpi↑] | Diapedesis |
| Intercellular adhesion molecule 1 | *Icam*1 | 2.29^1^ | 7.92E-06 | [7dpi↑-28dpi↑] | Diapedesis |
| Selectin, endothelial cell | *Sele* |  | Not detected |  | Diapedesis |
| Lama4 laminin, alpha 4 | *Lama*4 | -1.07^1^ | 2.47E-02 | [7dpi↓] | Neuroinvasion |
| Coagulation factor II receptor-like 1 | *F2rl*1 |  | Not detected |  | Brucipain mediated neuroinvasion |
| Guanine nucleotide binding protein | *Gnaq* | -4.63^1^ | 5.02E-08 | [7dpi↓] | Brucipain mediated neuroinvasion |
| Phospholipase C, beta 1 | *Plcb*1 |  | Not detected |  | Brucipain mediated neuroinvasion |
| Phospholipase C, beta 2 | *Plcb*2 |  | Subthreshold |  | Brucipain mediated neuroinvasion |
| Phospholipase C, beta 3 | *Plcb*3 | -1.50^6^ | 1.63E-05 | [7dpi↑] | Brucipain mediated neuroinvasion |
| Phospholipase C, beta 4 | *Plcb*4 | 1.61^5^ | 5.94E05 | [7dpi↓] | Brucipain mediated neuroinvasion |
| Protein kinase C, alpha | *Prkc*a | 1.23^1^ | 6.17E-04 | [7dpi↓] | Brucipain mediated neuroinvasion |
| Protein kinase C, beta | *Prkc*b | 2.58^6^ | 3.15E-07 | [7dpi↓] | Brucipain mediated neuroinvasion |
| Protein kinase C, gamma | *Prkc*g | -1.52^7^ | 7.94E-04 | [7dpi↑] | Brucipain mediated neuroinvasion |

b)

| Proposed Stage 2 Biomarker Genes | Symbol | Max FC^#^ | Adj p value | Pattern | Comment |
| --- | --- | --- | --- | --- | --- |
| Chemokine (C-X-C motif) ligand 10 | *Cxcl*10 | 8.72^4^ | 3.16E-05 | [0-28dpi↑] | T cell and trypanosome neuroinvasion |
| Chemokine (C-X-C motif) ligand 13 | *Cxcl*13 | 17.83^4^ | 9.86E-06 | [0-28dpi↑] | B cell migration |
| Intercellular adhesion molecule 1 | *Icam*1 | 2.85^4^ | 1.42E-05 | [7dpi↑-28dpi↑] | Super Ig CAM leukocyte-endothelial cell |
| Vascular cell adhesion molecule 1 | *Vcam*1 | 4.04^5^ | 1.07E-06 | [7dpi-28dpi↑] | Super Ig CAM leukocyte-endothelial cell |
| Beta-2-microglobulin | *B*2m | 8.04^4^ | 5.60E-05 | [28dpi↑] | Antigen processing and presentation. |
| Secreted phosphoprotein 1 | *Spp*1 | 2.34^5^ | 1.54E-04 | [7dpi↓] | Osteopontin cell matrix interaction |
| GTP cyclohydrolase 1 | *Gch*1 | 2.19^1^ | 1.69E | [7dpi↑] | Neopterin synthesizing enzyme |
| Lipocalin 2 | *Lcn*2 | 2.81^4^ | 4.21E-05 | [28dpi↑] | Amin et al [31] Secreted Biomarker |
| Secretory leukocyte peptidase inhibitor | *Slpi* | PCR^+ve^ |  | [28dpi↑] | Amin et al [31] Secreted Biomarker |
| Ceruloplasmin | *Cp* | 2.28^7^ | 1.77E-05 | [28dpi↑] | Amin et al [31] Secreted Biomarker |
| Granulin | *Grn* | 2.81^4^ | 1.43E-05 | [7dpi↑-28dpi↑] | Amin et al [31] Secreted Biomarker |
| Reelin | *Reln* | 2.04^7^ | 5.12E-06 | [7dpi↓] | Amin et al [31] Secreted Biomarker |
| Serglycin | *Srgn* | 1.52 | 1.37E-06 | [0-28dpi↑] | Amin et al [31] Secreted Biomarker |
| Translocator protein | *Tspo* | 3.08 | 9.84E-05 | [7dpi↑-28dpi↑] | Amin et al [31] Secreted Biomarker |
| Beta-2-microglobulin | *B*2m | 8.04^4^ | 5.60E-05 | [28dpi↑] | Amin et al [31] Secreted Biomarker |
| Serum amyloid A 3 | *Saa*3 | 26.4^4^ | 1.34E-06 | [28dpi↑] | Macrophage enriched (this study) |

Max FC^#^ denotes the Comparison^#^ with the maximum fold change.
