# Supplementary material for: Microarray profiling predicts early neurological and immune phenotypic traits in advance of CNS disease during disease progression in Trypanosoma. b. brucei infected CD1 mouse brains
Source: PLoS Negl Trop Dis. 2021 Nov 11;15(11):e0009892. doi: 10.1371/journal.pntd.0009892 (PMC8584711; doi:10.1371/journal.pntd.0009892)
Supplement: S10 Table — Pathogenic candidate genes, were selected based on a high FC, inferred biological significance or both, were grouped into 13 functional categories. Each gene was matched against its Comparison # with the maximum fold change (Max FC#), adj p value and expression pattern (Fig 3.). (DOCX) [file pntd.0009892.s015.docx]

**S10 Table. Candidate pathogenesis genes**.

| Gene | Symbol | Max FC^#^ | | Adj p value | | Pattern | Comment |
| --- | --- | --- | --- | --- | --- | --- | --- |
| Synaptic genes |  |  | |  | |  |  |
| Kinesin family member 5C | *Kif*5c | -8.43^1^ | | 8.47E-09 | | [7dpi↓] | Dopaminergic |
| GABA A receptor, subunit beta 3 | *Gabrb3* | -5.54^1^ | | 3.36E-08 | | [7dpi↓] | Serotonergic |
| GABA A receptor, subunit gamma 2 | *Gabrg*2 | -4.93^1^ | | 3.51E-08 | | [7dpi↓] | GABAergic |
| Synaptotagmin I | *Syt*1 | -3.41^1^ | | 1.03E-07 | | [7dpi↓] | Synaptic Vesicle Cycle |
| Synaptotagmin 4 | *Syt*4 | -4.92^1^ | | 2.35E-07 | | [7dpi↓] | Synaptic Vesicle Cycle |
| Synaptotagmin 16 | *Syt*16 | -2.77^1^ | | 7.39E-08 | | [7dpi↓] | Synaptic Vesicle Cycle |
| Ca/calmodulin-dependent Ser protein kinase | *Cask* | 2.75^5^ | | 4.63E-08 | | [7dpi↑] | Pre- and postsynaptic signalling |
| Neurexin I | *Nrxn*1 | 4.73^5^ | | 1.52E-08 | | [7dpi↓] | Synaptic CAM |
| Neurexin 3 | *Nrxn*3 | 4.43^6^ | | 3.52E-05 | | [7dpi↓] | Synaptic CAM |
| Cell adhesion molecule 1 | *Cadm*1 | 3.53^6^ | | 3.60E-07 | | [7dpi↓] | Synaptic CAM aka SynCam1 |
| Neuroligin 2 | *Nlgn*2 | -2.11^7^ | | 9.69E-07 | | [7dpi↑] | Synaptic CAM |
| CAM genes |  |  | |  | |  |  |
| Contactin 1 | *Cntn*1 | 6.18^6^ | | 8.47E-08 | | [7dpi↓] | CAM Axo-glial formation |
| Neuronal growth regulator 1 | *Negr*1 | 2.79^6^ | | 1.56E-06 | | [7dpi↓] | Neural growth factor |
| Neuronal cell adhesion molecule | *Nrcam* | 4.01^5^ | | 4.07E-08 | | [7dpi↓] | Neural development and myelination |
| Apoptotic genes |  |  | |  | |  |  |
| Baculoviral IAP repeat-containing 2 | *Birc*2 | -4.25^1^ | | 1.03E-07 | | [7dpi↓] | ApoCanD gene |
| Cytochrome c, somatic | *Cycs* | -2.91^1^ | | 4.69E-07 | | [7dpi↓] | ApoCanD gene |
| Apoptotic peptidase activating factor 1 | *Apaf*1 | 2.08^5^ | | 8.10E-05 | | [7dpi↓] | ApoCanD gene |
| Phosphoinositide-3-kinase regulatory subunit 1 | *Pik*3r1 | 5.51^5^ | | 1.97E-08 | | [7dpi↓] | *Apoptosis* ID^4210^ gene |
| Calpain 2 | *Capn*2 | -1.69^5^ | | 2.35E-04 | | [7dpi↓] | *Apoptosis* ID^4210^ gene |
| BCL2-associated X protein | *Bax* | -1.77^6^ | | 5.25E-06 | | [7dpi↑] | ApoCanD gene |
| BCL2-like 1 | *Bcl*2l1 | -1.79^6^ | | 2.19E-05 | | [7dpi↑] | ApoCanD gene |
| BCL2-associated agonist of cell death | *Bad* | -2.80^7^ | | 2.83E-07 | | [7dpi↑] | ApoCanD gene |
| Interleukin-1 receptor-associated kinase 2 | *Irak*2 | 1.71^6^ | | 2.16E-06 | | [7dpi↑] | *Apoptosis* ID^4210^ gene |
| Protein kinase cAMP dependent regulatory type I beta | *Prkar*1b | -2.16^7^ | | 9.35E-07 | | [7dpi↑] | *Apoptosis* ID^4210^ gene |
| Caspase 8 | *Casp*8 | 1.35^7^ | | 5.62E-04 | | [28dpi↑] | ApoCanD gene |
| Caspase 9 | *Casp*9 | -1.35^6^ | | 4.15e-04 | | [7dpi↑] | ApoCanD gene |
| Fas Ligand (TNF receptor superfamily member 6) | *Fasl* |  | | Subthreshold | | [7dpi↑-28dpi↑] | *African trypanosomiasis* ID^5143^ gene |
| Fas (TNF receptor superfamily member 6) | *Fas* | 1.84^7^ | | 1.01E-05 | | [0-28dpi↑] | *African trypanosomiasis* ID^5143^ gene |
| Fas (TNFRSF6)-associated via death domain | *Fadd* | 1.68^5^ | | 1.39E-05 | | [7dpi↑] | FASL-FAS Adaptor protein |
| Apoptotic peptidase activating factor 1 | *Apaf*1 | 2.08^5^ | | 8.10E-05 | | [7dpi↓] | ApoCanD gene |
| Phosphoinositide-3-kinase regulatory subunit 1 | *Pik*3r1 | 5.51^5^ | | 1.97E-08 | | [7dpi↓] | *Apoptosis* ID^4210^ gene |
| Calpain 2 | *Capn*2 | -1.69^5^ | | 2.35E-04 | | [7dpi↓] | *Apoptosis* ID^4210^ gene |
| BCL2-associated X protein | *Bax* | -1.77^6^ | | 5.25E-06 | | [7dpi↑] | ApoCanD gene |
| BCL2-like 1 | *Bcl*2l1 | -1.79^6^ | | 2.19E-05 | | [7dpi↑] | ApoCanD gene |
| BCL2-associated agonist of cell death | *Bad* | -2.80^7^ | | 2.83E-07 | | [7dpi↑] | ApoCanD gene |
| Interleukin-1 receptor-associated kinase 2 | *Irak*2 | 1.71^6^ | | 2.16E-06 | | [7dpi↑] | *Apoptosis* ID^4210^ gene |
| Protein kinase cAMP dependent regulatory type I beta | *Prkar*1b | -2.16^7^ | | 9.35E-07 | | [7dpi↑] | *Apoptosis* ID^4210^ gene |
| Caspase 8 | *Casp*8 | 1.35^7^ | | 5.62E-04 | | [28dpi↑] | ApoCanD gene |
| Caspase 9 | *Casp*9 | -1.35^6^ | | 4.15e-04 | | [7dpi↑] | ApoCanD gene |
| Fas Ligand (TNF receptor superfamily member 6) | *Fasl* |  | | Subthreshold | | [7dpi↑-28dpi↑] | *African trypanosomiasis* ID^5143^ gene |
| Fas (TNF receptor superfamily member 6) | *Fas* | 1.84^7^ | | 1.01E-05 | | [28dpi↑] | *African trypanosomiasis* ID^5143^ gene |
| Fas (TNFRSF6)-associated via death domain | *Fadd* | 1.68^5^ | | 1.39E-05 | | [7dpi↑] | FASL-FAS Adaptor protein |
| Heat shock protein genes |  |  | |  | |  |  |
| Heat shock protein 8 | *Hspb*8 | 2.49^1^ | | 7.84E-06 | | [7dpi↑] | Member of Hsp20 family |
| HSPA(heat shock 70kDa)binding protein cochaperone 1 | *Hspbp*1 | -2.69^6^ | | 9.04E-09 | | [7dpi↑] | Hsp 70 binding protein – chaperone activity |
| Heat shock protein 1A | *Hspa*1a | -2.14^5^ | | 4.77E-05 | | [7dpi↑] | Hsp 70 binding protein – chaperone activity |
| Heat shock protein 9 | *Hspa*9 | -5.05^1^ | | 3.24E-09 | | [7dpi↓] | Hsp 70 binding protein – chaperone activity |
| Heat shock protein 90, beta (Grp94), member 1 | *Hsp*90b1 | 7.43^7^ | | 5.88E-08 | | [7dpi↓] | Secretory protein chaperone |
| Heat shock protein 90, alpha class A member 1 | *Hsp*90aa1 | 5.44^6^ | | 9.98E-08 | | [7dpi↓] | Signalling protein chaperone |
| DnaJ heat shock protein family (Hsp40) member C4 | *Dnajc*4 | -2.01^7^ | | 4.15E-07 | | [7dpi↑] | HSP70 Co-chaperone |
| DnaJ heat shock protein family (Hsp40) member C12 | *Dnajc*12 | -1.56^6^ | | 4.77E-04 | | [7dpi↑] | HSP70 Co-chaperone |
| DnaJ heat shock protein family (Hsp40) member C17 | *Dnajc*17 | -1.34^6^ | | 1.32E-04 | | [7dpi↑] | HSP70 Co-chaperone |
| DnaJ heat shock protein family (Hsp40) member C30 | *Dnajc*30 | -1.87^6^ | | 4.05E-07 | | [7dpi↑] | HSP70 Co-chaperone |
| DnaJ heat shock protein family (Hsp40) member C2 | *Dnajc*2 | -3.78^1^ | | 1.39E-08 | | [7dpi↓] | HSP70 Co-chaperone |
| DnaJ heat shock protein family (Hsp40) member C6 | *Dnajc*6 | 3.16^5^ | | 1.39E-06 | | [7dpi↓] | HSP70 Co-chaperone |
| DnaJ heat shock protein family (Hsp40) member C7 | *Dnajc*7 | 3.88^5^ | | 3.78E-08 | | [7dpi↓] | HSP70 Co-chaperone |
| DnaJ heat shock protein family (Hsp40) member C10 | *Dnajc*10 | 4.26*^5^* | | 1.37E-08 | | [7dpi↓] | HSP70 Co-chaperone |
| Coiled-coil domain (Ccdc) gene family |  |  | |  | |  |  |
| Coiled-coil domain containing 124 | *Ccdc*124 | -4.70^7^ | | 5.77E-03 | | [7dpi↑] | Cytokinetic |
| Coiled-coil domain containing 181 | *Ccdc*181 | 2.06^1^ | | 6.27E-08 | | [7dpi↓] | Microtubule binding |
| Coiled-coil domain containing 85b | *Ccdc*85b | -2.06^5^ | | 3.18E-06 | | [7dpi↑] | Transcriptional repressor |
| Coiled-coil domain containing 55 | *Ccdc*55 | 2.23^6^ | | 1.44E-08 | | [7dpi↓] | Splicing |
| Coiled-coil domain containing 132 | *Ccdc*132 | -3.16^1^ | | 2.32E-08 | | [7dpi↓] | Recycling of internalizes transferrin receptor |
| Coiled-coil domain containing 174 | *Ccdc*174 | 3.06^5^ | | 1.92E-09 | | [7dpi↓] | Neuronal development |
| Protease genes |  |  | |  | |  |  |
| Cathepsin Z | *Cts*Z | 2.17^1^ | | 1.93E-06 | | [7dpi↑-28dpi↑] | Lysosomal cysteine protease cathespin |
| Cathepsin H | *Cts*H | 2.94^4^ | | 1.65E-03 | | [7dpi↑-28dpi↑] | Lysosomal cysteine protease cathespin |
| Cathepsin F | *Cts*F | -2.28^6^ | | 3.42E-07 | | [7dpi↑] | Lysosomal cysteine protease |
| Cathepsin A | *Cts*A | -3.20^6^ | | 1.78E-06 | | [7dpi↑] | Lysosomal serine protease cathespin |
| Cathepsin K | *Cts*K | -1.42^5^ | | 5.09E-04 | | [7dpi↑] | Lysosomal cysteine protease |
| Serine/cysteine peptidase inhibitor clade A member3G | *Serpina*3g | 11.66^4^ | | 2.41E-06 | | [28dpi↑] | Protease inhibitor BBB dysfunction |
| Serine/cysteine peptidase inhibitor clade A member3h | *Serpina*3h | 7.38^4^ | | 1.26E-03 | | [28dpi↑] | Protease inhibitor |
| Serine/cysteine peptidase inhibitor clade A member 3f | *Serpina*3f | 1.81^1^ | | 1.58E-02 | | [28dpi↑] | Protease inhibitor BBB dysfunction |
| Serine/cysteine peptidase inhibitor clade G member 1 | Serping1 | 5.87^4^ | | 3.26E-05 | | [28dpi↑] | Protease inhibitor BBB dysfunction |
| Histone genes |  |  | |  | |  |  |
| H2A clustered histone 7 | *H2ac*7 | 4.26^1^ | | 2.63E-07 | | [7dpi↑] | Core H2A histone |
| H2A clustered histone 24 | *H2ac*24 | 4.00^1^ | | 9.68E-08 | | [7dpi↑] | Core H2A histone |
| H2B clustered histone 12 | *H2bc*12 | -1.49^5^ | | 3.97E-04 | | [7dpi↑] | Core H2B histone |
| H3.3 histone B | *H3f*3b | 3.09^5^ | | 2.00E-07 | | [7dpi↓] | Core H3 histone |
| H4 clustered histone | *H4c*8 | 2.121 | | 1.23E-05 | | [7dpi↑] | Core H4 histone |
| Slc/Abc genes |  |  | |  | |  |  |
| Solute Carrier Family 6a Member 9 | *Slc*6a9 | -3.05^7^ | | 7.59E-07 | | [7dpi↑] | Slc6 Na-Cl dependant neurotransmitter family |
| Solute Carrier Family 6a Member 12 | *Slc*6a12 | -2.45^7^ | | 1.28E-06 | | [7dpi↑] | Slc6 Na-Cl dependant neurotransmitter family |
| Solute Carrier Family6a Member 13 | *Slc*6a13 | 2.94^6^ | | 1.69E-05 | | [7dpi↑] | Slc6 Na-Cl dependant neurotransmitter family |
| Solute Carrier Family 6a Member 7 | *Slc*6a7 | -1.90^7^ | | 2.35E-05 | | [7dpi↓] | Slc6 Na-Cl dependant neurotransmitter family |
| Solute Carrier Family 6a Member 15 | *Slc*6a15 | -2.88^1^ | | 3.71E-07 | | [7dpi↓] | Slc6 Na-Cl dependant neurotransmitter family |
| Solute Carrier Family 17a Member 6 | *Slc*17a6 | -8.85^1^ | | 1.67E-09 | | [7dpi↓] | Glutamatergic Synaptic Vesicle Cycle |
| Solute Carrier Family 10a Member 4 | *Slc*10a4 | -1.60^5^ | | 9.28E-05 | | [7dpi↓] | Cholinergic |
| ATP-binding cassette, sub-family A (ABC1), member 2 | *Abca*2 | -1.84^6^ | | 8.54E-06 | | [7dpi↑] | BBB enriched Neural development |
| ATP-binding cassette sub-family C member 5 | *Abcc*5 | -1.70^1^ | | 4.24E-05 | | [7dpi↓] | BBB enriched Organic anion pump |
| Solute Carrier Family 2a Member 1 | *Slc*2a1 | 2.07^1^ | | 5.57E-05 | | [7dpi↑] | BBB enriched Glut1 Carbohydrate transport |
| Solute Carrier Family 2a Member 13 | *Slc*2a13 | -5.06^1^ | | 2.08E-08 | | [7dpi↓] | BBB enriched Glut13 Carbohydrate transport |
| Solute Carrier Family 38a Member 2 | *Slc*38a2 | -2.19^1^ | | 1.38E-04 | | [7dpi↓] | BBB enriched GABAergic and Glutamatergic |
| Solute Carrier Family 38a Member 3 | *Slc*38a3 | 2.03^1^ | | 1.26E-06 | | [7dpi↑] | BBB enriched GABAergic and Glutamatergic |
| Solute Carrier Family 38a Member 5 | *Slc*38a5 | 3.10^1^ | | 3.52E-05 | | [7dpi↑] | BBB enriched GABAergic and Glutamatergic |
| Solute Carrier Family 1a Member 3 | *Slc*1a3^1^ | -5.26^1^ | | 1.34E-06 | | [7dpi↓] | BBB enriched EAAT1 synapse BBB enriched |
| Solute Carrier Family 02b Member 1 | *Slc*O2b1 | 1.72^1^ | | 4.99E-04 | | [7dpi↑-28dpi↑] | BBB enriched Organic anion |
| Solute Carrier Family 6a Member 9 | *Slc*6a9 | -3.05^7^ | | 7.57E-07 | | [7dpi↑] | BBB enriched Na-Cl neurotransmitter family |
| Solute Carrier Family 6a Member 6 | *Slc6*a6 | 1.57^6^ | | 2.81E-05 | | [7dpi↓] | BBB enriched GABA neurotransmission |
| Solute Carrier Family 8a Member 1 | *Slc*8a1 | -4.64^1^ | | 2.00E-08 | | [7dpi↓] | Na^+^/Ca^2+^ exchanger |
| Solute Carrier Family 24a Member 2 | *Slc*24a2 | -9.71^1^ | | 5.78E-09 | | [7dpi↓] | Na^+^(Ca^2+^/K^+^) exchanger |
| Solute Carrier Family 2a Member 1 | *Slc*2a1 | 2.07^1^ | | 5.57E-05 | | [7dpi↑] | Circadian Gene Database *Slc* gene |
| Solute Carrier Family 2a Member 6 | *Slc*2a6 | -1.73^6^ | | 2.54E-04 | | [7dpi↑] | Circadian Gene Database *Slc* gene |
| Solute Carrier Family 2a Member 8 | *Slc*2a8 | -1.86^6^ | | 1.74E-07 | | [7dpi↑] | Circadian Gene Database *Slc* gene |
| Solute Carrier Family 6a Member 17 | *Slc*6a17 | -2.17^6^ | | 1.62E-07 | | [7dpi↑]^1^ | Circadian Gene Database *Slc* gene |
| Solute Carrier Family 27a Member 1 | *Slc*27a1 | -1.92^7^ | | 5.24E-07 | | [7dpi↑] | Circadian Gene Database *Slc* gene |
| Solute Carrier Family 4a Member 2 | *Slc*4a2 | -2.25^7^ | | 6.72E-07 | | [7dpi↑] | Circadian Gene Database *Slc* gene |
| Solute Carrier Family 5a Member 3 | *Slc*15a3 | 2.36^4^ | | 4.80E-04 | | [7dpi↑-28dpi↑] | Proton oligopeptide Microglial enriched |
| Solute Carrier Family 11a Member 1 | *Slc*11a1 | 2.41^4^ | | 3.77E-04 | | [7dpi↑-28dpi↑] | Proton metal ion Microglial enriched |
| Circadian genes |  |  | |  | |  |  |
| Circadian locomotor output cycles kaput | *Clock* | -3.76^1^ | | 1.15E-08 | | [7dpi↓] | SCN enriched circadian clock gene |
| Period circadian clock 1 | *Per*1 | 2.62^1^ | | 2.20E-04 | | [7dpi↑] | SCN enriched circadian clock gene |
| Nuclear receptor subfamily 1, group D, member 1 | *Nr1d*1 | -2.01^7^ | | 9.74E-07 | | [7dpi↑] | SCN c enriched circadian clock gene |
| RAR-related orphan receptor alpha | *Rora* | 3.93^5^ | | 6.36E-08 | | [7dpi↓] | SCN enriched circadian clock r gene |
| Nuclear receptor subfamily 1, group D, member 2 | *Nr1d*2 | 3.36^6^ | | 9.67E-08 | | [7dpi↓] | SCN enriched circadian clock gene |
| Circadian associated repressor of transcription | *Ciart* | -1.65^7^ | | 1.23E-05 | | [7dpi↑] | Clock gene repressor |
| Glutamate receptor, ionotropic, AMPA1 (alpha 1) | *Gria*1 | -2.68^1^ | | 1.97E-08 | | [7dpi↓] | Entrainment Glut ionotropic receptor AMPA |
| Glutamate receptor, ionotropic, AMPA2 (alpha 2) | *Gria*2 | -10.25^1^ | | 1.68E-07 | | [7dpi↓] | Entrainment Glut ionotropic receptor AMPA |
| Glutamate receptor, ionotropic, AMPA3 (alpha 3) | *Gria*3 | -4.13^1^ | | 2.48E-08 | | [7dpi↓] | Entrainment Glut ionotropic receptor AMPA |
| Glutamate receptor, ionotropic, NMDA1 (zeta 1) | *Grin*1 | 2.63^1^ | | 2.66E-05 | | [7dpi↑] | Glut receptor, ionotropic, NMDA |
| Inositol 1,4,5-trisphosphate receptor 1 | *Itpr*1 | -6.27^1^ | | 3.16E-07 | | [7dpi↓] | Entrainment Calcium signalling |
| Mitogen-activated protein kinase 1 | Mapk1 | -3.62^1^ | | 1.20E-07 | | [7dpi↓] | Entrainment signalling |
| Guanylate cyclase 1, soluble, alpha 1 | Gucy1a3 | -3.48^1^ | | 1.85E-07 | | [7dpi↓] | Entrainment cGMP-PKG signalling |
| Secretogranin II | *Scg2* | 5.85^5^ | | 1.39E-7 | | [7dpi↓] | Non-clock SCN enriched |
| Regulator of G-protein signalling 16 | *Rgs*16 | -1.59^5^ | | 1.31E-02 | | [7dpi↑] | Non-clock SCN enriched |
| Dopamine receptor D1a | *Drd*1a | 1.42^7^ | | 8.46E-04 | | [28dpi↑] | Non-clock SCN enriched |
| Abelson helper integration site 1 | *Ahi*1 | -4.93^1^ | | 5.86E-08 | | [7dpi↓] | SCN enriched |
| Thymoma viral proto-oncogene 3 | *Akt*3 | -2.97^1^ | | 6.65E-08 | | [7dpi↓] | SCN enriched |
| Ubiquitin specific peptidase 29 | *Usp*29 | -4.00^1^ | | 6.98E-04 | | [7dpi↓] | SCN enriched |
| Arginine vasopressin-induced 1 | *Avp* | -2.08^5^ | | 2.99E-09 | | [7dpi↑] | Non-clock SCN enriched |
| Vasoactive intestinal polypeptide | *Vip* | -3.08^1^ | | 1.74E-06 | | [7dpi↓] | Non-clock SCN enriched |
| Gamma-aminobutyric acid A receptor, subunit beta 1 | *Gabr*b1 | -2.82^1^ | | 3.00E-07 | | [7dpi↓] | GABA receptor |
| Gamma-aminobutyric acid A receptor, subunit delta | *Gabr*d | -2.42^7^ | | 1.74E-07 | | [7dpi↑] | GABA receptor |
| Thimet oligopeptidase 1 | *Thop*1 | -2.52^7^ | | 1.21E-07 | | [7dpi↑] | *African trypanosomiasis* ID^5143^ gene |
| Pro-melanin-concentrating hormone | *Pmch* | -5.56^1^ | | 9.25E-07 | | [7dpi↓] | Melatonin – sleep promoting hormone |
| Hypocretin | *Hcrt* | -1.6^7^ | | 1.34E-04 | | [7dpi↑] | Orexin – awake promoting hormone |
| Tryptophan metabolism |  |  | |  | |  |  |
| Cysteine conjugate-beta lyase 1 | *Ccbl*1 | -1.91^7^ | | 1.07E-07 | | [7dpi↑] | Tryptophan metabolism |
| Indolethylamine N-methyltransferase | *Inmt* | 2.36^1^ | | 1.54E-05 | | [7dpi↑] | Tryptophan metabolism |
| Tryptophan hydroxylase 2 | *Tph*2 | 2.13^6^ | | 7.24E-05 | | [7dpi↓] | Tryptophan metabolism |
| Indoleamine 2,3-dioxygenase 1 | *Ido*1 | 1.40^7^ | | 3.08E-05 | | [28dpi↑] | *African trypanosomiasis* ID^5143^ gene |
| Hypoxia genes | | | | | | | |
| Haemoglobin alpha, adult chain 1 | *Hba-*a1 | 3.34^9^ | 2.23E-02 | | [7dpi↑-28dpi↑] | | Adult α globin |
| Haemoglobin, beta adult t chain | *Hbb-*bt | 3.44^9^ | 4.06E-02 | | [7dpi↑-28dpi↑] | | Adult β globin t chain |
| Haemoglobin, beta adult s chain | *Hbb-*bs | 3.38^9^ | 3.86E-02 | | [7dpi↑-28dpi↑] | | Adult β globin s chain |
| Haemoglobin, beta adult minor chain | *Hbb-b*2 | 1.42^9^ | 2.48E-01 | | [7dpi↑-28dpi↑] | | Adult β globin minor chain |
| Neuroglobin | *Ngb* | -1.72^7^ | 3.52E-07 | | [7dpi↑] | | Neuroprotective |
| Cytoglobin | *Cygb* | 2.64^1^ | 2.81E-06 | | [7dpi↑] | | Neuroprotective |
| Transferrin receptor | *Tfrc* | -6.98^1^ | 9.92E-09 | | [7dpi↓] | | Iron transcytosis |
| Myelin genes | | | | | | | |
| Proteolipid protein 1 | *Plp*1 | 10.76^6^ | 8.10E-09 | | [7dpi↓] | | Major myelin structural protein |
| Myelin-associated oligodendrocytic basic protein | *Mobp* | 4.08^5^ | 1.24E-08 | | [7dpi↓] | | Myelin sheath compaction |
| Oligodendrocyte myelin glycoprotein | *Omg* | 3.71^6^ | 6.10E-07 | | [7dpi↓] | | CAM integral for CNS myelination. |
| Myelin transcription factor 1-like | *Myt*1 | -4.60^1^ | 2.11E-07 | | [7dpi↓] | | Myelin transcription factor |
| Sphingomyelin phosphodiesterase 1, acid lysosomal | *Smpd*1 | -2.26^6^ | 2.25E-07 | | [7dpi↑] | | Sphingomyelin enzyme |

Max FC^#^ denotes the Comparison^#^ with the maximum fold change.

nd. mRNA not detected.
