# Supplementary material for: Microarray profiling predicts early neurological and immune phenotypic traits in advance of CNS disease during disease progression in Trypanosoma. b. brucei infected CD1 mouse brains
Source: PLoS Negl Trop Dis. 2021 Nov 11;15(11):e0009892. doi: 10.1371/journal.pntd.0009892 (PMC8584711; doi:10.1371/journal.pntd.0009892)
Supplement: S11 Table — Immune genes were selected based on a high FC, inferred biological significance or both, were grouped into nine immune functional categories. Each gene was matched against its Comparison # with the maximum fold change (Max FC#), adj p value and expression pattern (Fig 3.). (DOCX) [file pntd.0009892.s016.docx]

**S11 Table. Host immune gene response.**

| Gene | Symbol | Max FC^#^ | Adj p-value | Pattern | Comment |
| --- | --- | --- | --- | --- | --- |
|  | | | | | |
| Innate and adaptive gene markers |  |  |  |  |  |
| Transmembrane protein 119 | *Tmem*119 | 2.51^1^ | 5.73E-05 | [7dpi↑-28dpi↑] | Microglial specific |
| Sialic acid binding Ig-like lectin H | *Siglech* | 2.94^7^ | 1.86E-06 | [7dpi↓] | Microglial specific |
| Triggering receptor expressed on myeloid cells 2 | *Trem*2 | 2.01^1^ | 9.12E-04 | [7dpi↑-28dpi↑] | Microglial specific |
| Macrophage expressed gene 1 | *Mpeg*1 | 4.38^7^ | 3.56E-06 | [28dpi↑] | Macrophage enriched |
| Complement component 4B | *C*4*b* | 7.26^4^ | 4.84E-05 | [28dpi↑] | Macrophage enriched |
| AHNAK nucleoprotein (desmoyokin) | *Ahnak* | 3.50^7^ | 1.39E-07 | [7dpi↓] | Macrophage enriched |
| Glial fibrillary acidic protein | *Gfap* | 2.33^4^ | 5.07E-03 | [7dpi↑-28dpi↑] | Astrocyte |
| Myeloid differentiation primary response gene 88 | *Myd*88 | 1.99^1^ | 1.35E-03 | [7dpi↑-28dpi↑] | TLR9-MYD88 Innate cascade |
| Toll-like receptor 9 | *Tlr*9 | PCR^+ve^ |  | [28dpi↑] | TLR9-MYD88 Innate cascade |
| Toll-like receptor 2 | *Tlr*2 | 2.86^4^ | 1.36E-05 | [28dpi↑] | Binds MYD88 |
| Toll-like receptor 3 | *Tlr*4 | 1.24^7^ | 1.86E-05 | [28dpi↑] | Binds MYD88 |
| Nuclear factor kappa light polypeptide enhancer in B cells 2 | *Nfkb*2 | PCR^+ve^ |  | [28dpi↑] | TLR9-MYD88 Innate cascade |
| Histocompatibility 2, class II antigen A, beta 1 | *H2-Ab*1 | 23.99^4^ | 3.16E-05 | [28dpi↑] | Antigen processing and presentation |
| Histocompatibility 2, class II antigen A, alpha | *H2-Aa* | 2.63^4^ | 3.16E-05 | [28dpi↑] | Antigen processing and presentation |
| Histocompatibility 2, K region locus 2 | *H2-K*2 | 12.72^4^ | 1.30E-04 | [28dpi↑] | Antigen processing and presentation |
| Histocompatibility 2, class II, locus Mb1 | *H2-DMb*1 | 4.48^1^ | 1.84-04 | [28dpi↑] | Antigen processing and presentation |
| Proteasome subunit, beta type 8 (peptidase 7) | *Psmb*8 | 11.93^4^ | 1.56E-05 | [7dpi↑-28dpi↑] | Immunoproteasome |
| Proteasome subunit, beta type 8 (peptidase 2) | *Psmb*9 | 8.48^4^ | 1.46E-05 | [7dpi↑-28dpi↑] | Immunoproteasome |
| Proteasome subunit, beta type 10 | *Psmb*10 | 2.40^1^ | 1.47E-05 | [7dpi↑-28dpi↑] | Immunoproteasome |
| Complement 1, q subcomponent, α polypeptide | *C1q*a | 4.14^4^ | 2.17E-05 | [7dpi↑-28dpi↑] | Complement Microglial specific |
| Complement 1, q subcomponent, β polypeptide | *C1q*b | 5.67 | 4.09E-08 | [7dpi↑-28dpi↑] | Complement Microglial specific |
| Complement 1, q subcomponent, c polypeptide | *C1q*c | 5.84^4^ | 5.78E-05 | [7dpi↑-28dpi↑] | Complement Microglial specific |
| CD74 antigen | *Cd*74 | 44.94^4^ | 5.03E-07 | [28dpi↑] | MHC II Polypeptide Regulatory function |
| Beta-2-microglobulin | *B*2m | 8.04^4^ | 5.60E-05 | [28dpi↑] | Amin et al [38] Secreted Biomarker |
| Serum amyloid A 3 | *Saa*3 | 26.4^4^ | 1.34E-06 | [28dpi↑] | Macrophage enriched (this study) |
| Diapedesis/CAM genes |  |  |  |  |  |
| Intercellular adhesion molecule 2 | *Icam*2 | 2.29^1^ | 7.92E-06 | [7dpi↑-28dpi↑] | Super Ig CAM leukocyte-endothelial cell |
| Intercellular adhesion molecule 5 | *Icam*5 | -2.18^6^ | 3.35E-07 | [7dpi↑] | Super Ig CAM leukocyte-endothelial cell |
| Selectin platelet (p-selectin) ligand | Selplg | -2.56^5^ | 1.20E-05 | [7dpi↑-28dpi↑] | Binds SELP critical in initial leucocyte capture |
| Integrin alpha 7 | *Itga*7 | -1.70^7^ | 1.24E-04 | [7dpi↑] | Major laminin receptor |
| Integrin beta 4 | *Itgb*4 | -2.17^7^ | 1.56E-05 | [7dpi↑] | CAM Integrin |
| Integrin beta 5 | *Itgb*5 | 2.17 | 3.34E-06 | [7dpi↑] | CAM Integrin adhesion to vitronectin |
| A disintegrin & metallopeptidase domain 9 | *Adam*9 | 1.97^6^ | 6.59E-06 | [7dpi↓] | Angiogenesis |
| A disintegrin & metallopeptidase domain 17 | *Adam*17 | 2.20^7^ | 1.80E-06 | [7dpi↓] | TNF cleavage |
| A disintegrin & metallopeptidase domain 23 | *Adam*23 | 1.98^6^ | 1.74E06 | [7dpi↓] | May function as an integrin in the brain |
| A disintegrin & metallopeptidase domain | *Adam*15 | -1.83^6^ | 3.54E-05 | [7dpi↑] | Multifunctional |
| CD274 antigen | *Cd*274 | 8.65^4^ | 1.08E-04 | [28dpi↑] | Cell death ligand involved in T cell suppression |
| CD40 antigen | *Cd*40 | 2.25^4^ | 3.16E-05 | [28dpi↑] | TNF-family member 5 |
| CD86 antigen | *Cd*86 | 2.30^7^ | 1.70E-06 | [28dpi↑] | T lymphocyte proliferation |
| Immunoglobulin genes |  |  |  |  |  |
| Immunoglobulin kappa chain variable 8 (V8) | *Igk*-V8 | 1.74^7^ | 4.20E-04 | [28dpi↑]-late induction | Igk gene family |
| Immunoglobulin kappa chain variable 34 (V34) | *Igk*-V34 | 1.42^9^ | 1.31E-03 | [28dpi↑]-late induction | Igk gene family |
| Immunoglobulin joining chain | *Igi* | 1.15^7^ | 4.69E-04 | [28dpi↑]-late induction | Links IgM monomers |
| Marginal zone B and B1 cell-specific protein 1 | *Mzb*1 | 7.89^4^ | 4.30E-05 | [28dpi↑]-late induction | IgM assembly and secretion |
| Fc receptor, IgG, low affinity IV | *Fcgr*4 | 8.77^4^ | 2.32E-05 | [28dpi↑] | Fc gamma receptor gene |
| Fc receptor, IgG, low affinity III | *Fcgr*3 | 3.26^4^ | 1.67E-04 | [28dpi↑] | Fc gamma receptor gene |
| Fc receptor, IgG, low affinity IIb | *Fcgr*2b | 1.52^4^ | 6.98E-03 | [28dpi↑] | Fc gamma receptor gene |
| Cytokine genes | | | | | |
| Interferon alpha beta | *Ifnα/β* | 1.41^1^ | 3.57E-05 | [7dpi↑] | Type I Interferon response |
| Interferon beta 1 | *Ifn*β1 | PCR+ve |  | [7dpi↑] | Type I Interferon response |
| Interferon regulatory factor 3 | *Irf*3 | 1.81^1^ | 9.81E-07 | [7dpi↑] | Type I IFN response transcription factor |
| Interferon gamma | *Ifnγ* | 1.26^4^ | 6.24E-05 | [28dpi↑] | Type 2 Interferon response |
| Tumour necrosis factor alpha | *Tnf*α | PCR^+ve^ |  | [28dpi↑] | Pro-inflammatory Th1 Cytokine |
| Interleukin 1 alpha | *Il1*α | 1.17^7^ | 7.94E-03 | [28dpi↑] | Pro-inflammatory Th1 Cytokine |
| Interleukin 1 beta | *Il1*β | 2.27^4^ | 2.53E-04 | [28dpi↑] | Pro-inflammatory Th1 Cytokine |
| Interleukin 18 | *Il*18 | 1.59^7^ | 7.44E-04 | [28dpi↑] | Pro-inflammatory Th1 Cytokine |
| Caspase 1 | *Casp*1 | 6.97^7^ | 6.60E-08 | [28dpi↑] | Inflammasome Pro-inflammatory caspase |
| Caspase 4, apoptosis-related cysteine peptidase | *Casp*4 | 1.85^7^ | 1.46E04 | [28dpi↑] | Inflammasome Pro-inflammatory caspase |
| Transforming growth factor beta 1 | *Tgfb*1 | -1.68^5^ | 1.90E-05 | [7dpi↑-28dpi↑] | Th2 Cytokine |
| Interleukin 6 | *Il*6^nd^ | PCR^+ve^ |  | [28dpi↑] | Th2 Cytokine |
| Interleukin-10 | *Il*10^nd^ | PCR^+ve^ |  | [28dpi↑] | Th2 Cytokine |
| Interleukin 6 receptor alpha | *Il*6ra | 1.35^4^ | 6.88E-03 | [28dpi↑] | Th2 Cytokine receptor |
| Interleukin 10receptor alpha | *Il*10ra | 1.43^4^ | 7.22E-03 | [28dpi↑] | Th2 Cytokine receptor |
| Interleukin 15 | *Il*15 | 1.25^4^ | 1.68E-03 | [28dpi↑] | T cell and NK cell proliferation |
| Interleukin 34 | *Il*34 | -2.05^7^ | 1.79E-08 | [7dpi↑]^1^ | Microglia |
| Colony stimulating factor 1 receptor | *Csfr*1 | 1.97^9^ | 9.64E-03 | [7dpi↑-28dpi↑] | Receptor for ligand IL34 |
| IK cytokine | *Ik* | -4.59^1^ | 2.77E-07 | [7dpi↓] | Spliceosome factor MHC II inhibition |
| JAK-STAT activity |  |  |  |  |  |
| Janus kinase 2 | *Jak*2 | 3.10^7^ | 2.26E-08 | [7dpi↓] | Regulation of interferon mediated signalling |
| Janus kinase 3 | *Jak*3 | -1.22^5^ | 3.56E-04 | [7dpi↑-28dpi↑] | Regulation of interferon mediated signalling |
| Signal transducer and activator of transcription 1 | *Stat*1 | 5.59^4^ | 5.24E-04 | [28dpi↑] | Regulation of interferon mediated signalling |
| Signal transducer and activator of transcription 2 | *Stat*2 | 1.37^4^ | ns | [28dpi↑] | Regulation of interferon mediated signalling |
| Signal transducer and activator of transcription 3 | *Stat*3 | 2.16^4^ | 1.05E-03 | [7dpi↑-28dpi↑] | Regulation of interferon mediated signalling |
| IFNα/β ISGs |  |  |  |  |  |
| Interferon regulatory factor 1 | *Irf*1 | 10.90^4^ | 9.28E-06 | [28dpi↑] | Type I IFN response Transcription Factor |
| Interferon regulatory factor 2 | *Irf*2 | 1.58^4^ | 7.65E-03 | [28dpi↑] | Type I IFN response Transcription Factor |
| Interferon regulatory factor 3 | *Irf*3 | 1.81^1^ | 9.81E-07 | [7dpi↑] | Type I IFN response Transcription Factor |
| Interferon regulatory factor 5 | *Irf*5 | 1.29^1^ | 1.45E-03 | [7dpi↑-28dpi↑] | Type I IFN response Transcription Factor |
| Interferon regulatory factor 7 | *Irf*7 | 1.83^4^ | 6.45E-04 | [7dpi↑-28dpi↑] | Type I IFN response Transcription Factor |
| Interferon regulatory factor 8 | *Irf*8 | 1.65^4^ | 8.20E-04 | [28dpi↑] | Type I IFN response Transcription Factor |
| Interferon regulatory factor 9 | *Irf*9 | 3.44^4^ | 3.60E-05 | [28dpi↑] | STAT1:STAT2:IRF9 (ISGF3) Complex |
| Interferon induced transmembrane protein 1 | *Ifitm*1 | 7.74^4^ | 2.09E-06 | [7dpi↑-28dpi↑] | Type I IFN ISG Response |
| Interferon induced transmembrane protein 2 | *Ifitm*2 | 2.61^4^ | 8.24E-04 | [7dpi↑-28dpi↑] | Type I IFN ISG Response |
| Interferon induced transmembrane protein 3 | *Ifitm*3 | 7.41^4^ | 2.14E-07 | [7dpi↑-28dpi↑] | Type I IFN ISG Response |
| IFN-induced protein tetratricopeptide repeats 2 | *Ifit*2 | 4.45^7^ | 6.14E-08 | [28dpi↑] | Type I IFN ISG Response |
| IFN-induced protein with tetratricopeptide repeats 3 | *Ifit*3 | 7.19^7^ | 9.12E-04 | [28dpi↑] | Type I IFN ISG Response |
| IFNγ ISGs | | | | | |
| Immunity-related GTPase family M member 1 | *Irgm*1 | 9.39^4^ | 1.21E-05 | [28dpi↑] | p47GTPase |
| Immunity-related GTPase family M member 2 | *Irgm*2 | 16.99^4^ | 9.44E-06 | [28dpi↑] | p47GTPase |
| Immunity-related GTPase family M member 3 | *Irgm*3 | 28.81^4^ | 4.72E-06 | [28dpi↑] | p47GTPase induced in *T. gondi* infection |
| Interferon gamma inducible protein 47 | *Ifi*47 | 4.40^4^ | 2.42E-04 | [28dpi↑] | p47GTPase induced in *T gondi* infection |
| Guanylate binding protein 2 | *Gbp*2 | 24.13^4^ | 6.04E-06 | [28dpi↑] | p65-67GTPase induced in *T gondi* infection |
| Guanylate binding protein 3 | *Gbp*3 | 10.54^4^ | 1.19E-06 | [28dpi↑] | Type II IFN p65-67GTPase |
| Guanylate binding protein 5 | *Gbp*5 | 2.41^4^ | 3.32E-06 | [28dpi↑] | Type II IFN p65-67GTPase |
| Guanylate binding protein 7 | *Gbp*7 | 3.67^4^ | 1.98E-06 | [28dpi↑] | Type II IFN p65-67GTPase |
| Guanylate binding protein 10 | *Gbp*10 | 1.58^4^ | 4.62E-04 | [28dpi↑] | Type II IFN p65-67GTPase |
| GTPase, very large interferon inducible 1 | *Gvin* | 2.25^4^ | 1.01E-05 | [28dpi↑] | Very High MW Family – unknown function |
| Immunosuppression genes |  |  |  |  |  |
| CD274 antigen | *Cd*274 | 8.65^4^ | 1.51E-04 | [28dpi↑] | Type I IFN immune suppression |
| Poly (ADP-ribose) polymerase family, member 14 | *Parp*14 | 4.90^7^ | 5.68E-07 | [28dpi↑] | Type I IFN immune suppression |
| Tripartite motif-containing 21 | *Trim*21 | 1.98^4^ | 1.65E-03 | [28dpi↑] | Type I IFN immune suppression |
| Ubiquitin specific peptidase 18 | *Usp*18 | 6.23^4^ | 1.78E-5 | [28dpi↑] | Type I IFN immune suppression |
| Suppressor of cytokine signalling 3 | *Socs*3 | 2.59^4^ | 1.54E-04 | [7dpi↑-28dpi↑] | Suppression of cytokine signalling |
| Protein inhibitor of activated STAT3 | *Pias*3 | -1.72^7^ | 1.85E-06 | [7dpi↑] | Inhibition of STAT3 activity |
| Forkhead box P3 | *Foxp*3 | -1.83^6^ | 1.56E-05 | [7dpi↑] | Treg mediated immunosuppression |
| Chemokine genes | | | | | |
| Chemokine (C-C motif) ligand 2 | *Ccl*2 |  | Subthreshold | [28dpi↑] | Recruits monocytes, not neutophils |
| Chemokine (C-C motif) ligand 4 | *Ccl*4 | 3.98^4^ | 1.92E-06 | [28dpi↑] | Binds CCR5. NK cell attractant |
| Chemokine (C-C motif) ligand 5 | *Ccl5* | 21.13^4^ | 3.25E-06 | [28dpi↑] | Binds CCR5. Recruits leuckocytes |
| Chemokine (C-C motif) ligand 7 | *Ccl*7 | 2.05^4^ | 6.62E-04 | [28dpi↑] | Recruits monocytes and eosinophils |
| Chemokine (C-C motif) ligand 9 | *Ccl*9 |  | Subthreshold | [7dpi↑-28dpi↑] | Microglia/Macrophage enriched |
| Chemokine (C-C motif) ligand 19 | *Ccl*19 |  | Subthreshold | [7dpi↑-28dpi↑] | Chemotactic for naïve CD4 and CD8 T cells |
| Chemokine (C-C motif) receptor 5 | *Ccr*5 | 2.12^7^ | 1.53E-07 | [28dpi↑] | Receptor for Ligands CCL4 and CCL5 |
| Chemokine (C-X-C motif) ligand 1 | *Cxcl*1 |  | Subthreshold | [28dpi↑] | Neutrophil chemoattractant |
| Chemokine (C-X-C motif) ligand 9 | *Cxcl*9 | 34.58^4^ | 2.54E-06 | [28dpi↑] | IFNg induced |
| Chemokine (C-X-C motif) ligand 10 | *Cxcl*10 | 8.72^4^ | 3.16E-05 | [28dpi↑] | IFNg induced – trypanosome neuroinvasion |
| Chemokine (C-X-C motif) ligand 11 | *Cxcl*11 | PCR^+ve^ |  | [28dpi↑] | IFNg induced |
| Chemokine (C-X-C motif) receptor 3 | *Cxcr*3 | PCR^+ve^ |  | [28dpi↑] | Receptor for Ligands CXCL9, 10 and 11 |
| Chemokine (C-X-C motif) ligand 12 | *Cxcl*12 | 1.44^1^ | 3.44E-04 | [7dpi↑-28dpi↑] | T lymphocyte attractant |
| Chemokine (C-X-C motif) ligand 13 | *Cxcl*13 | 17.83^4^ | 9.86E-06 | [28dpi↑] | B cell chemoattractant |
| Chemokine (C-X-C motif) ligand 16 | *Cxcl*16 | 4.41^4^ | 3.90E-06 | [7dpi↑-28dpi↑] | Function of CXCL16/CXCR6 axis is unknown |
| Chemokine (C-X-C motif) ligand 14 | *Cxcl14* | -2.22^6^ | 7.54E-05 | [7dpi↑] | B cell specific chemoattractant |
| Chemokine (C-X3-C motif) ligand 1 | *Cx3cl*1 | -1.91^6^ | 6.90E-06 | [7dpi↑] | T cell, monocyte and NK cell chemoattractant |
| Chemokine (C-X3-C motif) receptor 1 | *Cx*3*cr*1 | 2.83^4^ | 2.17E-05 | [28dpi↑] | Receptor for CX3CL1 |

Max FC^#^ denotes the Comparison^#^ with the maximum fold change.
