# Supplementary material for: Microarray profiling predicts early neurological and immune phenotypic traits in advance of CNS disease during disease progression in Trypanosoma. b. brucei infected CD1 mouse brains
Source: PLoS Negl Trop Dis. 2021 Nov 11;15(11):e0009892. doi: 10.1371/journal.pntd.0009892 (PMC8584711; doi:10.1371/journal.pntd.0009892)
Supplement: S12 Table — Candidate genes were designated a) as “disruptive” involving structural changes to the paracellular barrier complex or b) “functional” where a change in the activity of a range of non-cytokine mediators. Each gene was matched against its Comparison # with the maximum fold change (Max FC#), adj p value and expression pattern (Fig 3.). (DOCX) [file pntd.0009892.s017.docx]

**S12 Table. Genes associated with BBB integrity/impairment.**

| Gene | Symbol | MaxFC^#^ | Adj p value | Pattern | Comment |
| --- | --- | --- | --- | --- | --- |
| a) DISRUPTIVE CHANGES | | | | | |
| Ancillary paracellular barrier genes | | | | | |
| Endothelial cell-specific adhesion molecule | *Esam* | 2.36^4^ | 2.18E-06 | [7dpi↑] | JAM protein - Enriched in BBB |
| Cadherin associated protein alpha 1 | *Ctnna*1 | -2.68^1^ | 8.16E-06 | [7dpi↓] | Tight Junction ID^4530^ Catenin association with AJ complex |
| Cadherin associated protein alpha 2 | *Ctnna*2 | -2.96^1^ | 9.00E-06 | [7dpi↓] | Tight Junction ID^4530^ Catenin association with AJ complex |
| Spectrin beta, non-erythrocytic 1 | *Sptbn*1 | 2.78^5^ | 7.39E-07 | [7dpi↓] | Tight Junction ID^4530^ Interacts with Calmodulin in Ca^2+^manner |
| Myosin, heavy polypeptide 10, non-muscle | *Myh*10 | 2.92^5^ | 3.58E-07 | [7dpi↓] | Tight Junction ID^4530^ Regulation of cytoskeleton |
| Shroom family member 2 | *Shroom*2 | -2.22^1^ | 2.61E-06 | [7dpi↓] | Tight Junction ID^4530^ Endothelial morphology |
| Exocyst complex component 4 | *Exoc*4 | 2.20^5^ | 2.61E-7 | [7dpi↓] | Tight Junction ID^4530^ Exocytic vesicle : plasma membrane fusion |
| Tight junction associated protein 1 | *Tjap*1 | 1.64^1^ | 4.41E-07 | [7dpi↑] | Tight Junction ID^4530^  Ancillary structural protein |
| LLGL2 scribble cell polarity complex component | *Ligl*2 | -1.86^7^ | 3.69E-07 | [7dpi↑] | Tight Junction ID^4530^ Cell polarity |
| Actinin alpha 2 | *Actn*2 | -1.64^5^ | 9.43E-05 | [7dpi↑] | Tight Junction ID^4530^ Actin cross linker |
| Actin, beta | *Actb* | 1.71^1^ | 1.30E-03 | [7dpi↑-28dpi↑] | Tight Junction ID^4530^Actin cytoskeleton |
| Matrix metallopeptidase 2 | *Mmp*2 | -1.64^5^ | 4.31E-05 | [7dpi↑-28dpi↑] | ECM mediated BBB breakdown |
| Matrix metallopeptidase 9 | *Mmp*9 | -1.18^7^ | 1.51E-02 | [7dpi↑] | ECM mediated BBB breakdown |
| b) FUNCTIONAL CHANGES |  |  | |  | |
|  |  |  | |  |  |
| Neuroinvasion genes |  |  |  |  |  |
| Laminin, alpha 4 | *Lama*4 | -1.07^1^ | 2.47E-02 | [7dpi↓] | Trypanosome retention in endothelial perivascular space |
| Laminin, alpha 5 | *Lama*5 | -1.18^6^ | 1.28E-03 | [7dpi↑] | Trypanosome migration to brain parenchyma |
| Guanine nuc’tide binding protein, α q polypeptide’ | *Gnaq* | -4.63^1^ | 5.02E-08 | [7dpi↓] | Signal-transducing G protein |
| Phospholipase C beta 3 | *Plcb*3 | -1.50^6^ | 1.63E-05 | [7dpi↑] | Prod’n of the 2y messengers DAG and inositol 1,4,5-PO_4t_ |
| Phospholipase C beta 4 | *Plcb*4 | 1.61^5^ | 5.94E05 | [7dpi↓] | Prod’n of the 2y messengers DAG and inositol 1,4,5-PO_4t_ |
| Protein kinase C alpha | *Prkca* | 1.23^1^ | 6.17E-04 | [7dpi↓] | Response to production of diacylglycerol (DAG) |
| Protein kinase C-beta | *Prkcb* | 2.58^6^ | 3.15E-07 | [7dpi↓] | Response to production of diacylglycerol (DAG) |
| Protein kinase C gamma | *Prkcg* | -1.52^7^ | 7.94E-04 | [7dpi↑] | Response to production of diacylglycerol (DAG) |
| Calcium homeostasis genes |  |  |  |  |  |
| Ca^2+^ channel voltage-dependent β 4 subunit | *Cacn*b4 | -5.39^1^ | 1.88E-09 | [7dpi↓] | Calcium signalling |
| Ca^2+^/calmodulin-dependent protein kinase IV | *Camk*4 | -8.23^1^ | 8.47E-09 | [7dpi↓] | Calcium signalling |
| Endothelin receptor type B | *Ednrb* | -5.57^1^ | 1.52E-08 | [7dpi↓] | Calcium signalling |
| Guanine nucleotide binding protein α q | *Gnaq* | -4.63^1^ | 5.02E-08 | [7dpi↓] | Bruipain mediated BBB Breakdown/ Calcium signalling |
| Inositol 1,4,5-trisphosphate receptor 1 | *Itpr*1 | -6.27^1^ | 3.16E-07 | [7dpi↓] | Calcium signalling |
| ATPase Ca^2+^transporting cardiac muscle twitch 2 | *Atp*2a2 | -3.84^1^ | 4.93E-07 | [7dpi↓] | Calcium signalling |
| Phosphodiesterase 1A, calmodulin-dependent | *Pde*1a | 3.61^5^ | 6.29E-07 | [7dpi↓] | Calcium signalling |
| Protein phosphatase 3 catalytic subunit alpha | *Pp*3ca | 3.13^6^ | 3.31E-07 | [7dpi↓] | Calcium signalling |
| Calmodulin 2 | *Calm*2 | 2.66^6^ | 2.78E-05 | [7dpi↓] | Calcium signalling |
| Solute Carrier Family24a Member2 | *Slc*24a2 | -9.71^1^ | 5.78E-09 | [7dpi↓] | Na^+^(Ca^2+^/K^+^) exchanger |
| Solute Carrier Family8a Member1 | *Slc*8a1 | -4.64^1^ | 2.00E-08 | [7dpi↓] | Na^+^/Ca^2+^ exchanger |
| Angiogenic mediators |  |  |  |  |  |
| Vascular endothelial growth factor A | *Vegf*a | 1.69^6^ | 1.82E-04 | [7dpi↓] | Angiogenic factor - promotes BBB breakdown |
| Vascular endothelial growth factor B | *Vegf*b | -1.73^7^ | 2.26E-06 | [7dpi↑] | Angiogenic factor – endothelia proliferation |
| Platelet derived growth factor, B polypeptide | *Pdgf*b | -2.18^6^ | 1.49E-07 | [7dpi↑] | Angiogenic factor – BBB integrity |
| Angiopoietin 1 | *Angpt*1 |  | Subthreshold | [28dpi↑] | Angiogenic factor Ligand for tyrosine kinase receptor |
| Angiopoietin 2 | *Angpt*2 | 1.47^7^ | 6.32E-05 | [28dpi↑] | Angiogenic factor Vascular remodeling |
| TEK receptor tyrosine kinase | *Tek* | -1.42^1^ | 1.79E-04 | [7dpi↓] | Angiopoietin receptor |
| Angiopoietin-like-4 | *Angpt*4 | -1.91^7^ | 5.23E-04 | [7dpi↑] | Angiogenic factor |
| Angiopoietin-like 6 | *Angptl*6 | -2.05^7^ | 1.43E-06 | [7dpi↑] | Angiogenic factor |
| Angiogenic factor G patch & FHA domains 1 | *Aggf1* | 3.07^7^ | 1.65E-07 | [7dpi↓] | Angiogenic factor |
| Endoglin | *Eng* | 2.17^1^ | 7.75E-08 | [7dpi↑-28dpi↑] | β (TGFβ) associated receptor Angiogenic factor |
| Wingless-type Member 7a | *Wnt*7a | -2.77^7^ | 1.82E-07 | [7dpi↑] | Wnt lsignalling ligand |
| Wingless-type Member 7b | *Wnt*7b | -2.89^7^ | 1.68E-06 | [7dpi↑] | Wnt signalling ligand |
| Wingless-type Member 2 | *Wnt*2 | -1.78^7^ | 3.97E-06 | [7dpi↑] | Wnt signalling ligand |
| Wingless-type Member 4 | *Wnt*4 | -1.86^6^ | 4.71E-06 | [7dpi↑] | Wnt signalling ligand |
| Frizzled class receptor | *Fzd*2 | 1.88^1^ | 7.70E-05 | [7dpi↑] | Wnt ligand receptor |
| Miscellaneous mediators |  |  |  |  |  |
| Caveolin 1, caveolae protein | *Cav*1 | 1.86^6^ | 3.04E-04 | [28dpi↑] | Endothelial transcytosis |
| Plasmalemma vesicle –associated protein | *Plvap* | -1.93^6^ | 4.37E-03 | [7dpi↑] | Endothelial transcytosis |
| Major facilitator superfamily domain 2A | *Mfs*d2 | 1.68^7^ | 3.09E-03 | [7dpi↑] | Endothelial transcytosis |
| Lymphocyte antigen 6 complex, locus A | *Ly*6a | 9.22^4^ | 1.21E-05 | [28dpi↑] | Endothelial transcytosis |
| Aquaporin 4 | *Aqp*4 | 5.47^7^ | 3.16E-07 | [7dpi↓] | Enriched in astroglial end-feet |

Max FC^#^ denotes the Comparison^#^ with the maximum fold change.
